# Transcriptomic Profiling Reveals Novel Candidate Genes and Signalling Programs in Breast Cancer Quiescence and Dormancy †

**DOI:** 10.3390/cancers13163922

**Published:** 2021-08-04

**Authors:** Lewis A. Quayle, Amy Spicer, Penelope D. Ottewell, Ingunn Holen

**Affiliations:** 1 Department of Oncology and Metabolism, Medical School, University of Sheffield, Beech Hill Road, Sheffield S10 2RX, UK; amy.spicer-hadlington@crick.ac.uk (A.S.); p.d.ottewell@sheffield.ac.uk (P.D.O.); i.holen@sheffield.ac.uk (I.H.); 2 The Francis Crick Institute, Midland Road, London NW1 1AT, UK

**Keywords:** breast cancer, dormancy, quiescence, RNA-Seq, transcriptomics

## Abstract

**Simple Summary:**

Breast cancer can return many years after treatment of the primary tumour. This is caused by cells that have spread to other parts of the body and entered a non-dividing state called quiescence or dormancy that can last for decades. Dormant cancer cells are not sensitive to conventional chemo- and radiotherapies, which primarily target fast growing cells, and so can eventually resume growth to cause formation of tumours at secondary sites. The exact processes by which cancer cells become dormant are currently poorly understood. This study describes the use of model systems specifically developed to compare the genes used by dormant and dividing breast cancer cells, allowing the identification of a number of genes and cellular mechanisms that might underpin breast cancer cell dormancy and therefore represent promising novel candidates to inform development of new treatments to prevent breast cancer recurrence.

**Abstract:**

Metastatic recurrence, the major cause of breast cancer mortality, is driven by reactivation of dormant disseminated tumour cells that are defined by mitotic quiescence and chemoresistance. The molecular mechanisms underpinning mitotic quiescence in cancer are poorly understood, severely limiting the development of novel therapies for removal of residual, metastasis-initiating tumour cells. Here, we present a molecular portrait of the quiescent breast cancer cell transcriptome across the four main breast cancer sub-types (luminal, HER2-enriched, basal-like and claudin-low) and identify a novel quiescence-associated 22-gene signature using an established lipophilic-dye (Vybrant^®^ DiD) retention model and whole-transcriptomic profiling (mRNA-Seq). Using functional association network analysis, we elucidate the molecular interactors of these signature genes. We then go on to demonstrate that our novel 22-gene signature strongly correlates with low tumoural proliferative activity, and with dormant disease and late metastatic recurrence (≥5 years after primary tumour diagnosis) in metastatic breast cancer in multiple clinical cohorts. These genes may govern the formation and persistence of disseminated tumour cell populations responsible for breast cancer recurrence, and therefore represent prospective novel candidates to inform future development of therapeutic strategies to target disseminated tumour cells in breast cancer, eliminate minimal residual disease and prevent metastatic recurrence.

## 1. Introduction

Metastatic recurrence is the leading cause of mortality in advanced breast cancer. This process involves reactivation of disseminated tumour cells (DTCs) that colonise distal sites early during disease pathogenesis and remain clinically undetectable for a protracted time period before emerging to initiate secondary tumour growth [1]. Latent recurrence in breast cancer can be classified as both medium-term (2–5 years) or long-term (≥5 years) and is dependent on several independent risk factors, including primary tumour volume, tumour stage and oestrogen receptor (ER) status [2]. The median time to recurrence for patients with oestrogen receptor-negative (ER−) tumours is approximately 2 years, while more than 50% of relapses in oestrogen receptor-positive (ER+) cases occur 5 years or more after primary tumour diagnosis and surgical resection [3,4]. However, the 15-year recurrence and mortality rates for both ER− and ER+ breast cancer sub-types are comparable in patients diagnosed at early stages of disease [5]. These observations indicate both broad heterogeneity in recurrence patterns and potential commonality in the biology governing mitotic kinetics in DTC populations involved in latent relapse across breast cancer sub-types.

The phenomenon by which DTCs or micro-metastases remain at undetectable levels for prolonged latency periods is referred to as dormancy. Evidence from pre-clinical modelling and limited surgical samples indicate that tumour cell dormancy is the result of mitotic quiescence: a state of reversible or intermittent postponement of active cell cycle transition [6]. This quiescent state confers an innate ability of DTCs to survive conventional adjuvant treatments that primarily target proliferating cells [7,8]. Quiescent cancer cells have also been shown to actively regulate survival signalling mechanisms post-therapy, demonstrating that they do not merely survive therapeutic insult in a passive manner [9]. The consequent inability of conventional anti-cancer therapy to eliminate quiescent DTCs is a major barrier to development of curative treatments for latent metastatic disease that results in eventual progression and death in approximately 20% of breast cancer patients [10]. A greater understanding of the processes governing tumour cell quiescence therefore holds the key to development of anti-dormancy therapies required to prevent metastatic relapse.

Although obtaining quiescent DTCs from patient material remains unfeasible, a wide variety of pre-clinical models for identification, isolation and analysis of quiescent tumour cells now exist [6,11,12]. Amongst these, the use of lipophilic fluorochromes for pulse-chase tracking of cancer cell mitotic kinetics is one of the most affordable, reliable and easily implemented. The relative simplicity of this dye-retention method should not belie its effectiveness and potential; it has been successfully used to isolate and study quiescent, putative metastasis-initiating cells or DTCs in several cancers, including brain, breast, colon, ovarian, pancreatic, prostate and skin cancers [8,13,14,15,16,17,18,19]. The quiescent cells identified in these studies exhibit multiple proposed traits of DTCs, including enhanced metastatic and tumorigenic potential, chemo- and radio-therapy resistance, and morphogenetic characteristics associated with epithelial-mesenchymal-transition (EMT) and niche cell mimicry. Despite increased accessibility and affordability of gene- and protein-level profiling technologies, such as RNA sequencing (RNA-Seq) and reverse phase protein arrays (RPPA), most studies have not progressed beyond basic model validation and functional characterisation of isolated quiescent cells, thereby failing to realise the full potential for discovery of quiescence-associated molecular programmes.

We have developed a highly reliable Vybrant^®^ DiD retention model for identification, isolation and characterisation of mitotically quiescent tumour cell populations with potential to elucidate molecular mechanisms associated with tumour cell quiescence and dormant disease [8]. Here, we apply this model for isolation and whole-transcriptomic profiling of quiescent tumour cells from four distinct breast cancer sub-types and go on to identify a series of genes and functional programmes over-represented in both established and predicted quiescence-associated ontologies that correlate strongly with late recurrence (≥5 years after primary tumour diagnosis) in clinical breast cancer datasets. These genes represent sets of prospective novel targets for further unravelling the biology of quiescent DTCs and may provide future candidates for therapeutic strategies for patients with latent metastatic breast cancer.

## 2. Materials and Methods

### 2.1. Cell Culture

Fully authenticated MCF-7, SK-BR-3, MDA-MB-468 and MDA-MB-231 human breast cancer cell lines, sourced directly from the American Type Culture Collection (ATCC) (Manassas, VA, USA.) were maintained in RPMI-1640 basal medium (11 mM glucose, 2 mM L-glutamine) supplemented with 10% (*v/v*) foetal bovine serum (FBS) (Life Technologies Ltd., Paisley, UK).

### 2.2. Fluorescent Dye Labelling, Dye Retention Assays and Cell Sorting

Vybrant^®^ DiD labelling was performed in suspension according to the manufacturer’s instructions (Molecular Probes MP22885). Labelled samples were grown as adherent cultures for six passages post-staining. Cytofluorimetric analyses and fluorescence activated cell sorting (FACS) were undertaken as previously described using the BD™ LSR-II™ and BD™ FACSAria™ platforms (Beckton, Dickenson and Co. Plc., Oxford, UK), respectively [8].

### 2.3. RNA Extraction, Sequencing Library Preparation and mRNA Sequencing

Total RNA was isolated from DiD− and DiD+ cells at passage six post-labelling using the miRNeasy Micro Kit (Qiagen UK, Manchester, UK). NEBNext^®^ Ultra II Directional RNA Library Prep Kit for Illumina^®^ (NEB E7760S/L) and NEBNext Poly(A) mRNA Magnetic Isolation Module (NEB E7490) were used for preparation of sequencing libraries (New England Biolabs (UK) Ltd., Hitchin, UK). In total, 200 ng of DNA-free total RNA was used as input for each experimental sample and PCR undertaken during cDNA synthesis was for 12 cycles. NEBNext^®^ Multiplex Oligos for Illumina^®^ were used as index primers in accordance with the manufacturer−specified instructions (NEB E7335S/L). Sequencing was undertaken on the Illumina HiSeq™ 2500 platform in rapid run mode using the Illumina HiSeq™ Rapid Cluster Kit (Illumina, Inc., San Diego, CA, USA).

### 2.4. Bioinformatic and Statistical Analyses

FASTQ files were quality checked using FastQC v0.11.8 [20]. Samples were filtered for low quality reads (Phred quality score <20) and adapters removed using Cutadapt v2.6 [21]. Quality controlled reads were then aligned to the Human reference genome assembly GRCh38.p13 using STAR aligner v2.7.0f [22]. Quantification of transcripts was performed using RSEM v1.3.1. [23]. All downstream processing of transcript count data, statistical analyses and graphical modelling of data were performed in R v4.0.3.

Testing for differential expression of genes was conducted using the quasi-likelihood functionality of edgeR v3.32.1 [24]. A model matrix was constructed specifying the experimental design, linear model coefficients and parameterisation used for differential expression analysis. Lowly expressed genes were filtered by retaining those with count per million (CPM) read values above *k* in *n* samples, where *k* was determined by sample library sizes and *n* by the design matrix. Library size normalisation was undertaken using the Trimmed Mean of M-values (TMM) method. Negative binomial dispersions (common, trended and gene-wise) were estimated by weighted likelihood empirical Bayes method before a quasi-likelihood negative binomial generalised log-linear model was fitted to the data. Gene-wise testing for differential expression was undertaken according to the contrasts specified by the design matrix relative to a defined fold-change threshold. Differentially expressed genes were detected with a log_2_ fold-change significantly above 1.5 (up-regulated) or below −1.5 (down-regulated) at a false-discovery rate (FDR) cut-off of 5%.

Principal component analysis (PCA) and unsupervised agglomerative hierarchical clustering on the basis of Euclidean distance computed with an average-linkage matrix were performed using TMM normalised, log_2_-transformed CPM values. Gene set enrichment analysis (GSEA) was performed for gene sets retrieved from Molecular Signatures Database (MSigDB) using fgsea v1.16.0 [25] and msigdbr v7.2.1 [26], respectively. A composite functional association network was constructed, visualised and topologically analysed with igraph v1.2.6 [27] using interaction data downloaded from GeneMANIA [28] and STRING [29] databases. Self-loops and weightless edges were removed and duplicate edges aggregated according to their mean weight during network construction. Centrality metrics calculated during topological analysis included total degree, betweenness, eigenvalue, and closeness. A weighted centrality score for each network node was computed by multidimensional scaling (where dimensionality of the reconstructed space (*k*) = 1) followed by log_10_ transformation.

Publicly available cell line data were downloaded from Broad Institute Cancer Cell Line Encyclopaedia (CCLE). Clinical data sets were downloaded from the Gene Expression Omnibus and the MSKCC Cancer Genomics Data Server using GEOquery v2.58.0 [30] and cgdsr v1.3.0 [31], respectively. All gene level expression data used in composite data sets were normalised and log_2_ transformed prior to integration using the ComBat algorithm in sva v3.38.0 to remove dataset-specific bias [32]. Optimised one-to-one mapping between gene and probe sets was achieved for microarray data using jetset v3.4.0 [33]. Summarised expression of multiple genes as a single, integrated signature score was achieved by calculating the standardised (mean-centred and scaled) difference between geometric means of the normalised and log_2_-transformed expression values for all up- and down-regulated genes prior to subsequent analysis.

Kaplan–Meier survival curves were estimated using survival v3.2.7 [34] and survminer v.0.4.8 [35]. Cohorts were dichotomised into “high” and “low” signature score expression groups based on the optimal stratification point. This was determined using survivALL v0.9.3 [36] to iteratively calculate every possible point of stratification for the cohort (n−1) when ranked in increasing order of expression value and selecting the cut-off with the most-significant corresponding FDR-adjusted *p*-value. For exhaustive Cox regression analyses, this was done for all genes in the input gene list and the summed log_2_ hazard ratio for all significant stratification points (FDR-adjusted *p*-value ≤ 0.05) normalised to the total rank (cohort) size for each gene calculated. These values were adjusted automatically for significant covariates in multivariate analyses.

Univariate statistical comparisons between two groups were performed in R v4.0.3 using the Wilcoxon rank-sum test (Mann–Whitney U test), as indicated, where appropriate, in the respective figure legend. Statistical significance was attributed when a *p*-value of ≤0.05 was obtained.

## 3. Results

### 3.1. Fluorescent Dye-Retaining Sub-Clones as a Model of Breast Cancer Cell Quiescence

**Figure 1:**
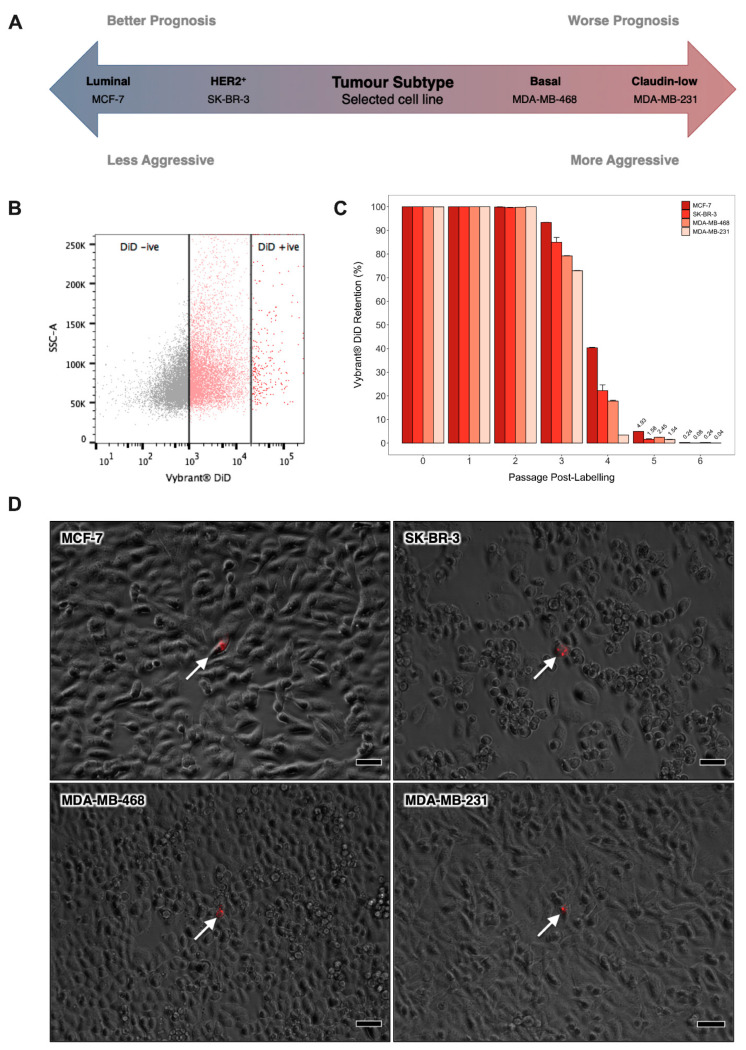
Identification of Quiescent Breast Cancer Cells by Lipophilic Dye Retention In vitro. (**A**) Schematic depicting the prognosis of the main breast tumour subtypes and the representative cell line selected to model each of these. (**B**) Cytofluorimetric dot plot illustrating the gating strategy for identification of Vybrant^®^ DiD retaining cells in adherent human breast cancer cell cultures after six passages of culture growth. Cytofluorimetric platforms were calibrated at the outset of each experiment using a Vybrant^®^ DiD negative (DiD−) control sample and a Vybrant^®^ DiD positive (DiD+) control sample that had been freshly labelled with Vybrant^®^ DiD. (**C**) Proportion of label-retaining cells within adherent MCF-7, SK-BR-3, MDA-MB-468 and MDA-MB-231 cultures over six consecutive passages of culture growth as measured by flow cytometry. Data are expressed as mean ± SE (n = 3). (**D**) Phase-contrast and fluorescent image overlays of MCF-7, SK-BR-3, MDA-MB-468 and MDA-MB-231 cultures after 6 passages of culture growth post-staining with Vybrant^®^ DiD (scale bar = 50μm). White arrows indicate DiD+ cells (red).

In order to facilitate identification of molecular programmes central to breast cancer cell quiescence irrespective of molecular sub-type, we expanded our established in vitro model system to incorporate a panel of cell lines that broadly represent the main types of human breast tumour; MCF-7 (luminal), SK-BR-3 (HER2-enriched), MDA-MB-468 (basal-like) and MDA-MB-231 (claudin-low) (Figure 1A). Vybrant^®^ DiD was progressively diluted from cultures over sequential passages to reveal a dye-retaining, quiescent sub-population. After six passages, the mean number of DiD− cells exceeded 50%, while DiD+ cells accounted for less than 0.5% of the total population in all cultures (Figure 1B,C). At this point, dye-retaining DiD+ cells were distinguishable from cells that had lost their initial DiD label, or cells that retained only a low level of DiD, as assessed by flow cytometry and fluorescence microscopy (Figure 1B,D). These data were consistent with our previous report that DiD retention is inversely correlated with net mitotic activity and pilot studies indicating latent tumourigenicity of DID+ cells in vivo (data not shown) [8].

### 3.2. Transcriptomic Profiling of Dye-Retaining Sub-Clones Identifies Quiescence-Associated Biological Programmes

**Figure 2:**
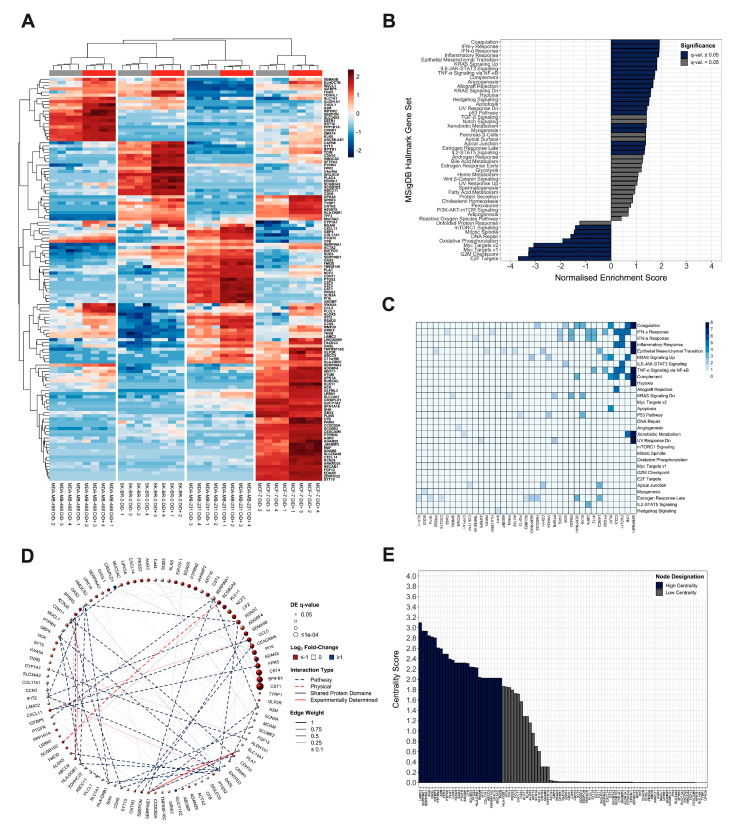
Ontological and Functional Analysis of the Quiescent Breast Cancer Cell Transcriptome. (**A**) Agglomerative hierarchically clustered heatmap of mean-centered, TMM normalised log_2_-transformed CPM gene expression values as quantified by mRNA-Seq. Clustering is unsupervised and based on Euclidean distances calculated from an average-linkage matrix. All genes detected as differentially expressed with a log_2_ fold-change significantly above 1.5 (up-regulated) or below −1.5 (down-regulated) at an FDR-adjusted *p*-value (*q*-value) ≤ 5% across all breast cancer cell lines are shown (127 total). Grey and red bars represent Vybrant^®^ DiD−negative (DiD−) and Vybrant^®^ DiD− positive (DiD+) grouping, respectively. (**B**) Gene set enrichment analysis of the differentially expressed genes using MSigDB hallmark collection gene ontologies denoted by enrichment score normalised to mean enrichment of randomised equivalent samples (hypergeometric test with post-hoc Benjamini-Hochberg FDR adjustment). (**C**) Analysis of leading edge genes identified as driving significant enrichment of MSigDB hallmark collection gene sets. Columns indicate quiescence-associated signature genes contributing to the enrichment signal of the gene set(s) indicated by row labels. Colour is scaled to the number of leading edge gene sets in which each gene appears. For clarity, only signature genes that appear in ≥1 ontologies are shown. (**D**) Composite functional association network constructed for genes detected as differentially regulated with a log_2_ fold-change significantly above 1.5 (up-regulated) or below −1.5 (down-regulated) at an FDR-adjusted *p*-value (*q*-value) ≤ 5% across all four breast cancer cell lines. For clarity, only empirically determined (non-inferred) network members are shown. Vertex (circle) size is proportional to FDR-adjusted *p*-value of differential regulation (*q*-value); node colour is proportional to the log_2_ fold-change between proliferative and quiescent breast cancer cells. Connecting edge (line) colour and type represents interaction relationship. Edge thickness is proportional to linear regression–derived network weighting. (**E**) Topological analysis of the quiescence-associated signature gene network. Centrality score represents the log_10_ transformed combined centrality metrics (total degree, betweenness, eigenvalue, and closeness) calculated during topological analysis. Genes with combined centrality scores in the upper−quartile (75th percentile) are designated as “high centrality” while those that failed to meet this cut-off as “low centrality”.

As we aimed to identify differentially regulated genes between quiescent and proliferative breast cancer cells, irrespective of molecular sub-type, total RNA was extracted from the DiD− and DiD+ fractions of each cell line and subjected to whole-transcriptomic mRNA-Seq. Differential expression testing and unsupervised dimensionality reduction analyses revealed a tendency of DiD− and DiD+ cells to cluster separately, with a notable degree of reproducibility between biological replicates (Figure 2A, Supplementary Figure S1A) and distinctive cell-type specific profiles of differentially regulated genes (Figure 2A, Supplementary Figures S1B and S2). When all data sets were analysed together, 127 genes were detected as differentially expressed between quiescent and proliferating cells with a log_2_ fold-change significantly above 1.5 or below −1.5 at a FDR cut-off of 5% (123/127 up-regulated and 4/127 down-regulated), irrespective of molecular sub-type (Figure 2A). Approximately 90% of these differentially regulated genes (113/127) are reported to correlate with metastasis (Supplementary Table S1). Ontological enrichment analysis showed that the quiescent breast cancer cell transcriptome was significantly enriched with genes involved in epithelial-mesenchymal transition (EMT), extracellular matrix (ECM) interaction/degradation, immunoregulation and stress-tolerance-related signalling (e.g., hypoxia and KRAS) (Supplementary Figure S3A). These data are consistent with both reported and consensus predicted traits of quiescent breast cancer cells in the context of dissemination, tumour initiation and mitotic quiescence. In addition, the most strongly negatively enriched pathways all pertained to reduced mitotic activity (Figure 2B, Supplementary Figure S3A). Analysis of the leading-edge genes driving significant enrichment profiles in GSEA revealed 19 genes of high biological interest due to their contribution to the enrichment signal of multiple (≥2) gene sets (Figure 2C). Of these, 89% (17/19) have previously been associated with metastasis, of which approximately 25% (4/17) have been correlated with quiescence or dormancy in cancer (Supplementary Table S1).

To comprehensively understand the functional context within which the differentially regulated quiescence-associated gene products may interact, a composite functional association network was constructed, integrating information from multiple sources collated in the GeneMANIA and STRING databases (Figure 2D). Validation analyses showed that the average quiescence-associated network clustering coefficient was significantly greater than those of networks generated by 10^4^ rounds of degree-preserving randomised edge rewiring events (*p* = 2.2 × 10^−16^, two-tailed one-sample *Z*-test, Supplementary Figure S3B), and that inferred network members were enriched within the quiescence-associated transcriptome (NES = 1.91, *p* = 1.04 × 10^−5^, Supplementary Figure S3C). These results importantly showed that differentially expressed quiescence-associated gene products have a greater propensity to form a network of connected protein groups (modules) than would be expected by random chance alone. Subsequent network topological analysis revealed 28 proteins that were highly linked to neighbouring nodes (weighted centrality scores in the 75th percentile), suggesting that they may participate in one or more sub-networks of functionally related proteins (Figure 2E, Supplementary Table S1). In support of this, network community detection analysis identified a total of 20 sub-network communities with ≥3 members; ~80% (22/28) of genes identified as being of high biological interest based on their centrality metrics were represented in the four largest, most highly interconnected networks (Supplementary Table S1). As expected, there was a significant proportional overlap between genes identified as being of high biological interest by leading edge analysis and those identified by network analyses (*p* = 0.000488, Fisher’s exact test), and functional themes included EMT, ECM adhesion and remodelling, inflammation and immune regulation. Collectively, these analyses identify a panel of genes relating to cancer metastasis and/or dormancy, highlight strongly connected interactors and common functional associations between these genes, and suggest the roles that these genes and ontologies might play in underpinning the biology of quiescent breast cancer cells.

### 3.3. Quiescence-Associated Gene Expression Correlates with Low Proliferative Activity and Late Breast Cancer Recurrence

In order to ascertain the potential prognostic value of the 127 quiescence-associated genes, a composite data set of 572 patients (CB572, Supplementary Table S2) was constructed from three gene expression profiling data sets (GSE11276, GSE2034 and GSE2603) widely used to validate experimentally determined genes and signatures implicated in metastasis and dormancy in breast cancer [37,38,39,40,41]. Critically, these data sets contain primary tumour ER status, distant metastatic event indicators and distant metastasis-free survival (DMFS) follow-up times of at least 7 years. They also contain sufficient cumulative patient numbers to facilitate statistically viable comparisons between early and late distant metastatic recurrence events (<5 and ≥5 years after primary tumour diagnosis, respectively).

**Figure 3:**
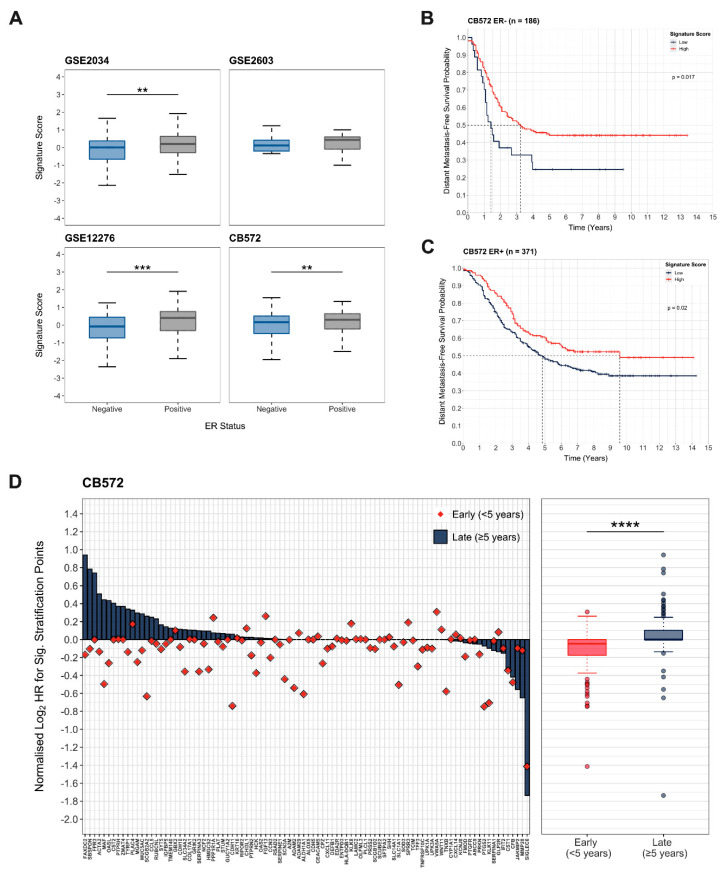
Quiescence-Associated Genes are Correlated with Late Metastatic Recurrence. (**A**) Comparison of integrated expression scores for quiescence-associated genes in patients with oestrogen receptor-positive (ER+) and oestrogen receptor-negative (ER−) tumours within data sets (GSE2034, GSE2603 and GSE12276) comprising the CB572 cohort (Wilcoxon rank-sum test, ** = *p* ≤ 0.01, *** = *p* ≤ 0.001). (**B**) Distant metastasis-free survival probability over time in patients within the CB572 cohort with ER− tumours integrated signature scores for expression of quiescence-associated signature genes (n = 186, log-rank test). (**C**) Distant metastasis-free survival probability over time in patients within the CB572 cohort with ER+ tumours stratified by integrated signature scores for expression of quiescence-associated signature genes (n = 371, log-rank test). (**D**) Cox proportional hazards survival analysis of the quiescence-associated signature genes represented in primary breast tumours of patients in the CB572 composite data set (n = 572). Patients were stratified at all possible cut-off points (n−1) for all signature genes. For each gene, the log_2_-transformed cumulative hazard ratio for all cut-off points significantly (*p* ≤ 0.05) associated with late distant metastatic recurrence and early distant metastatic recurrence (<5 and ≥5 years after primary tumour diagnosis, respectively) normalised to total possible cut-off points are shown. Quiescence-associated signature genes were significantly associated with late metastatic recurrence (Wilcoxon rank-sum test, **** = *p* ≤ 0.0001).

As ER status is an established independent risk factor determining latency of metastatic recurrence in breast cancer [2], we initially established the relationship between ER status and the integrated expression score for the quiescence-associated gene set. There was a significant positive correlation between ER status and signature score in 2 out of 3 of the CB572 data sets (Figure 3A). More importantly, the integrated expression scores were significantly higher in ER+ compared to ER− tumours in the CB572 cohort (*p* = 0.00298, Wilcoxon rank-sum test, Figure 3A), reflecting the longer latency period generally associated with ER+ breast cancer.

To provide an indication of the correlation between expression of quiescence-associated genes and late DMFS, survival analysis was conducted using the Kaplan–Meier model to estimate time to recurrence when stratified by quiescence-associated signature score within the ER− and ER+ sub-sets of the CB572 cohort. In both cases, high expression of quiescence-associated genes was associated with a significant increase in the median time to metastatic recurrence. In patients with ER− tumours, the median time to distant recurrence increased from approximately 18 months to over 3 years (*p* = 0.0170, log-rank test, Figure 3B), while in patients with ER+ tumours, this was extended from approximately 5 years to 9.5 years (*p* = 0.0200, log-rank test, Figure 3C). These data suggest that high expression of the quiescence-associated gene set identified is associated with later metastatic recurrence, irrespective of ER status.

To more comprehensively assess the prognostic value of the 127 quiescence-associated gene set, an exhaustive multivariate Cox regression analysis approach was used, with ER status as a covariate [36]. This enabled quantification of the overall association between the quiescence-associated signature and risk of early or late distant recurrence, and facilitated identification of individual genes with potential prognostic importance, all while accounting for any additional variance resulting from ER status. The results demonstrated that a significantly greater number of quiescence signature genes are associated with an increased risk of late metastatic recurrence (*p* = 3.31 × 10^−8^, Wilcoxon rank-sum test, Figure 3D). This association was further validated in a second independent breast cancer cohort (CB482, Supplementary Table S2) composed of three further microarray gene expression profiling data sets (GSE1456, GSE6532 and GSE7930) that met all inclusion criteria as detailed for CB572 (*p* = 4.62 × 10^−5^, Wilcoxon rank-sum test, Supplementary Figure S3D). Taken together, these data indicate that high expression of quiescence-associated signature genes within primary tumours correlates more strongly with late, compared to early, distant breast cancer recurrence.

**Figure 4:**
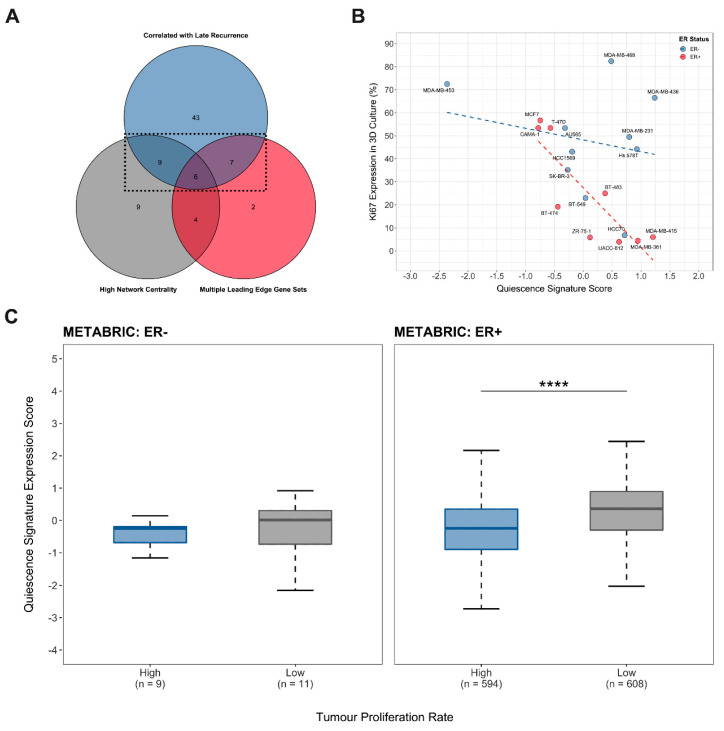
Quiescence-Associated Signature Gene Expression is Elevated in Oestrogen Receptor-Positive Breast Cancers with Low Proliferative Activity. (**A**) Venn diagram illustrating selection of the 22 quiescence-associated signature genes based on having both high functional and prognostic significance in gene set enrichment or network topological analysis and proportional hazards regression analysis. (**B**) Correlation analysis of quiescence-associated signature expression scores for breast cancer cell lines with their respective published proliferation indices determined by Ki67 expression in 3D culture [42]. Oestrogen receptor-negative (ER−) cell lines (shown in blue, Spearman’s rank correlation coefficient *ρ* = −0.0545, *p* = 0.90) and oestrogen receptor-positive (ER+) cell lines (shown in red, Spearman’s rank correlation coefficient *ρ* = −0.787, *p* = 0.01) are plotted independently. (**C**) Comparison of integrated expression score for the 22 gene quiescence-associated signature between patient tumours with high and low proliferation rates (pre-determined by Memorial Sloan Kettering Cancer Centre using the ERα, HER2 and AURKA three-gene classifier) in ER+ and ER− METABRIC cohorts (Wilcoxon rank-sum test, **** = *p* ≤ 0.0001).

Finally, the outcomes of the leading-edge gene set analysis, functional association network analysis and proportional-hazards survival modelling were combined to identify a condensed quiescence-associated gene signature with both high functional and prognostic value. This final 22-gene signature includes genes that appeared in multiple leading-edge gene sets driving the enrichment of MSigDB Hallmark ontologies, were identified as being highly interactive during network topological analysis and that were significantly associated with late metastatic recurrence in CB572 and/or CB482 data sets (Figure 4A, Table 1). To assess the potential predictive value of this signature, correlative regression analysis was conducted to determine the association between the integrated 22-gene signature expression score and the inherent proliferative capacity (relative proportional Ki67 expression in 3D mammosphere culture) reported for 19 human breast cancer cell lines [42] (Figure 4B). No statistically significant correlation between the Ki67 proliferation index and signature score was found for ER− cell lines, but there was a strong negative correlation for ER+ cell lines (Spearman’s rank correlation coefficient *ρ* = −0.055, *p* = 0.90 and Spearman’s rank correlation coefficient *ρ* = −0.79, *p* = 0.01, respectively). This suggested that the quiescence-associated signature identified was indicative of a program that imposes slower growth kinetics on ER+ tumour cells that may, in turn, lead to quiescence in ER+ tumours in vivo. In order to establish whether this was the case, the integrated expression score for the 22 quiescence-associated signature genes was compared between patient tumours with high and low proliferation rates in ER− and ER+ strata of the METABRIC cohort (Figure 4C). As predicted, the results showed no significant difference between the mean gene signature expression scores for fast and slow growing ER− tumours (Wilcoxon rank-sum test, *p* = 6.03 × 10^−1^) but a strongly significant increase in the mean expression of the 22-gene signature in slow growing ER+ tumours (Wilcoxon rank-sum test, *p* = 1.38 × 10^−23^). Taken together, these findings show that high expression of the quiescence-associated 22-gene signature correlates with both low proliferative activity and with late metastatic recurrence or dormant disease in breast cancer.

## 4. Discussion

Here, we present a molecular portrait of the quiescent breast cancer cell transcriptome across molecular sub-types, as well as a novel 22-gene quiescence-associated signature in breast cancer. These data were developed by extracting highly differentially regulated genes from the combined expression profiles of Vybrant^®^ DiD−retaining quiescent sub-clones isolated from four breast cancer sub-types in vitro. The gene expression profiles of these populations, both individually and collectively, exhibited up-regulation of genes with well-characterised roles in halting cell cycle progression (e.g., CDKN1A and IGFBP5), as well as displaying ubiquitous negative enrichment for hallmark gene sets involved in transcriptional regulation of proliferation, cell cycle transition and mitosis [43,44]. Both the differentially regulated set of 127 quiescence-associated genes and gene sets positively enriched within the broader quiescent breast tumour cell transcriptome incorporate elements of established dormancy-instructive programmes, such as hypoxia, TGF-β, p38 and p53 signalling [45]. These findings are in agreement with our previous demonstration of the quiescent nature of dye-retaining cells, indicating that the transcriptional programmes associated with this population might represent elements of novel molecular mechanisms involved in metastatic breast cancer dormancy [8]. Correlative analyses using publicly available clinical breast cancer data sets support this hypothesis; the median time to distant metastatic recurrence was found to be significantly longer in patients with tumours displaying a high overall quiescence-associated gene signature scores than those who exhibited lower scores. Furthermore, a significantly greater proportion of quiescence-associated genes possessed hazard ratios indicative of increased probability of late metastatic recurrence. Although promising indicators, these represent correlative associations, and it will be imperative to establish mechanistic causal links between individual genes, or sets of genes, described here and cellular quiescence or tumoural dormancy using pharmacological or genetic means (e.g. siRNA). The functional association programmes we have identified provide useful starting points for such studies. Establishing such a mechanistic link could pave the way for development of targeted therapies to maintain the activity of dormancy-instructive genes, thereby prolonging quiescence and retaining disease at a chronic state. Early reports of the successful development of an effective specific agonist of the tumour dormancy-regulating orphan nuclear receptor NR2F1 have provided an exciting indication of the potential for such targeted therapeutics [46]. The inhibition of key survival genes or pathways could also potentially enable specific removal dormant tumour cells before they resume growth and progression, thereby reducing the risk of recurrence while avoiding disruption of normal physiological processes such as haematopoiesis and tissue regeneration that rely on cellular quiescence.

The transcriptomes of quiescent breast cancer cells were not only enriched for programmes governing proliferative activity but were also significantly enriched for genes associated with purported features of metastasis-initiating DTCs; stemness, morphogenetic signalling and EMT, ECM interaction/degradation, angiogenic regulation and xenobiotic metabolism [12,45,47]. A large proportion of these genes have previously been associated with metastasis, further supporting the relevance of the gene set identified in defining the metastatic potential of tumour cells. We have previously reported that dye-retaining breast cancer cells exhibit de novo chemoresistance and subsequent clonal outgrowth in vitro, as well as elevated activity of the stemness marker and drug metabolising enzyme family aldehyde dehydrogenase (ALDH) [48,49], a member of which (ALDH1A1) was also found to be significantly up-regulated across all breast cancer types here. These findings collectively indicate that the dye-retaining population consists of cells with enhanced metastatic potential; data from a closely related study in prostate cancer has previously demonstrated that dye-retaining cells are significantly more metastatic in vivo compared to their rapidly dividing counterparts [18]. Going forward it will be important to comprehensively establish whether dye-retaining breast cancer cells of various molecular sub-types are able to form tumours in vivo, whether they display enhanced capacity to engraft at distal sites, and how the temporal course of tumourigenesis and spread compares with that of the proliferative cell population.

It is well established that cellular signalling cascades that result in tumoural dormancy are subject to reciprocal crosstalk between DTCs and their microenvironment [1,12,45,47]. The quiescence-associated programmes elucidated from the dye-retention model in vitro do not account for any signal reprogramming or interactions that occur within the metastatic niche in vivo. What at first glance could appear to be a deficiency of the model system may be exploited to dissect dormancy-instructive programmes and establish how they are influenced by microenvironmental interactions. Although comparable systems have generated gene signatures with considerable prognostic utility in breast cancer [50], it is crucial to recognise the absence of this added layer of signalling complexity and the potential role of reciprocal interplay between the tumour cell intrinsic quiescence programmes identified here and metastatic niche constituents in vivo.

Finally, although neither molecular sub-type nor clinical data were used to refine our quiescence-associated gene signature, patients with ER+ breast cancers were found to have significantly higher integrated signature scores for expression of these genes than patients with ER− tumours. While the median time to distant metastatic recurrence was significantly extended in patients with high gene signature expression scores in both ER− and ER+ sub-groups, the overall time to distant recurrence was considerably longer in patients with high signature scores in ER+ tumours, compared with ER− tumours. The former observation suggests the potential for more comprehensive meta-analyses, or extension of the dye-retention model to more discrete cell line panels to develop specific gene signatures that could reflect sub-type-specific quiescence mechanisms. The latter observation likely reflects the established general clinical tendency for ER+ tumours to recur later compared with ER− tumours [2]. Given the established specific organotropism of breast cancer sub-types [51], it may also be possible to deconvolute the gene signature to identify sub-sets associated with dormancy in a metastatic site-specific manner. In addition, a number of established quiescence mechanisms are conserved across cancer types; for example, increased p38:ERK signalling ratio, NR2F1 and p27 have all been shown to be dormancy-inductive in head and neck squamous cell carcinoma, breast and prostate cancers [52,53,54,55,56]. On this basis, we can also speculate that elements of our quiescence signature might be extended to other medium- or long-latency cancers such as colon and prostate cancer.

## 5. Conclusions

In summary, we report a novel quiescence-associated breast cancer gene signature extracted from whole-transcriptomic profiling of quiescent sub-clones across breast cancer cell lines of distinct molecular sub-types. The genes within this signature show a significant degree of functional and clinical relevance to breast cancer metastasis and dormancy. Further investigation of these genes and the functional interactors we have identified might yield novel targets for therapies enabling elimination of the cells responsible for dormant cancer and late recurrence, thereby improving outcomes for patients.

## Figures and Tables

**Table 1:** Quiescence-Associated Genes with High Functional and Prognostic Significance.

Name	HUGO Symbol	Entrez ID	Ensembl ID	Log_2_ Fold-Change ^a^	DE *q*-Value ^b^	MSigDB Hallmark Gene Set Leading Edges ^c^	Centrality Score ^d^	Centrality Designation ^e^	Correlation with Late Recurrence ^f^	Metastasis Associated ^g^	Quiescence or Dormancy Associated ^h^
C-C motif chemokine ligand 5	CCL5	6352	ENSG00000271503	2.75622625	6.40 × 10^−7^	5	1.726356886	Low	CB572 + CB482	+	+
cellular communication network factor 3	CCN3	4856	ENSG00000136999	1.159158799	0.003129394	0	2.369572457	High	CB572 + CB482	+	
cadherin 11	CDH11	1009	ENSG00000140937	0.924409835	0.001008869	2	0.017134571	Low	CB572 + CB482	+	+
collagen type XVII alpha 1 chain	COL17A1	1308	ENSG00000065618	1.159975436	0.002670111	0	2.021417131	High	CB572 + CB482	+	
formyl peptide receptor 3	FPR3	2359	ENSG00000187474	2.954316158	3.43 × 10^−8^	0	2.487201841	High	CB572	+	
3-hydroxy-3-methylglutaryl-CoA synthase 2	HMGCS2	3158	ENSG00000134240	1.36731073	0.000805151	2	0.004347429	Low	CB572 + CB482	+	
insulin like growth factor binding protein 5	IGFBP5	3488	ENSG00000115461	1.115110029	0.00391699	0	2.801887967	High	CB572 + CB482	+	
kallikrein related peptidase 8	KLK8	11202	ENSG00000129455	1.33535789	7.97 × 10^−5^	3	1.277111171	Low	CB482	+	
maltase-glucoamylase	MGAM	8972	ENSG00000257335	1.391777411	0.042802637	0	2.039476442	High	CB572 + CB482	+	
mucin 5AC, oligomeric mucus/gel-forming	MUC5AC	4586	ENSG00000215182	1.422900799	0.000379937	0	2.021250734	High	CB572	+	+
2’-5’-oligoadenylate synthetase like	OASL	8638	ENSG00000135114	1.151350653	0.023043447	2	2.819995388	High	CB572 + CB482		
plasminogen activator, tissue type	PLAT	5327	ENSG00000104368	1.053223792	0.031200647	4	2.391649253	High	CB572	+	
prostaglandin F receptor	PTGFR	5737	ENSG00000122420	1.228190238	0.004757316	2	2.315990672	High	CB482	+	
prostaglandin-endoperoxide synthase 2	PTGS2	5743	ENSG00000073756	1.429349462	0.022239393	3	2.233337779	High	CB482	+	
protein tyrosine phosphatase receptor type H	PTPRH	5794	ENSG00000080031	1.065756445	0.001072713	0	2.487144917	High	CB572	+	
protein tyrosine phosphatase receptor type N2	PTPRN2	5799	ENSG00000155093	1.878824513	2.86 × 10^−5^	0	2.607454659	High	CB572	+	
radical S-adenosyl methionine domain containing 2	RSAD2	91543	ENSG00000134321	1.765109846	8.21 × 10^−7^	3	0.021323383	Low	CB572 + CB482	+	
serpin family A member 1	SERPINA1	5265	ENSG00000197249	1.203836437	7.93 × 10^−6^	3	2.928302569	High	CB482	+	
serpin family A member 3	SERPINA3	12	ENSG00000196136	1.255309782	0.00051306	2	0.008651795	Low	CB572	+	
serpin family E member 1	SERPINE1	5054	ENSG00000106366	1.423828199	0.013356874	8	2.933522416	High	CB572	+	
TNF receptor superfamily member 10c	TNFRSF10C	8794	ENSG00000173535	1.136170418	0.013356874	0	2.021292344	High	CB482	+	
von Willebrand factor A domain containing 5A	VWA5A	4013	ENSG00000110002	1.412217075	0.001530294	2	0.00434743	Low	CB482		

^a^ Log_2_-transformed fold change in mean normalised gene transcript counts as determined by RNA-Seq (proliferating Vybrant^®^ DiD- versus quiescent Vybrant^®^ DiD+ sub-populations), ^b^ False discovery rate adjusted *p*-value of differential expression according to comparison of mean normalised gene transcript counts, ^c^ Number of times the corresponding gene was found in the leading edge gene set driving the statistically significant enrichment of one of the MSigDB Hallmark gene sets analysed in GSEA, ^d^ Centrality score calculated as the log_10_-transformed weighted sum of total degree, betweenness, eigenvalue, and closeness centrality metrics combined by multidimensional scaling, ^e^ Designation as having a “high” or “low” centrality score metric relative to the 75th percentile cut-off value (centrality score ≥ 2), ^f^ Indication of which clinical data set(s) in which the corresponding gene was found to have a statistically significant association with late metastatic recurrence (≥5 years after primary tumour diagnosis) by Cox regression analysis, ^g^ Association with metastasis according to automated mining of PubMed^®^ using the search “gene[Title/Abstract] AND metasta*[Title/Abstract]”, ^h^ Association with dormancy or quiescence in cancer according to automated mining of PubMed^®^ combining the results of searches “gene[Title/Abstract] AND quiescen*[Title/Abstract] AND cancer[Title/Abstract]” or “gene[Title/Abstract] AND dorman*[Title/Abstract] AND cancer[Title/Abstract]”.

## Data Availability

All data analyses undertaken in the R language and programming environment were using open-source packages and the functions contained therein, as detailed in the methods section. The raw data that support the findings of this study are available from the corresponding author upon reasonable request. Clinical gene level expression and follow-up data analysed in this study are already publicly available and their accession numbers given in the main body of the text and Supplementary Table S2.

## Supplementary Materials

The following are available online at https://www.mdpi.com/article/10.3390/cancers13163922/s1, Figure S1: Cell Line-Specific Transcriptomic Analysis of Quiescent and Proliferative Sub-Populations (A) Principal component analysis of the whole transcriptomic profiles of proliferative Vybrant^®^ DiD−negative (DiD−) and quiescent Vybrant^®^ DiD−positive (DiD+) sub-populations in MCF-7, SK-BR-3, MDA-MB-468 and MDA-MB-231 human breast cancer cell lines. The first two principal components account for >50% of the total variance in all datasets. (B) Volcano plots of genes quantified in the DiD− and DiD+ transcriptomes within MCF-7, SK-BR-3, MDA-MB-468 and MDA-MB-231 human breast cancer cell lines. Vertical lines represent the log_2_ fold-change thresholds for detection of significantly differentially expressed genes at the FDR-adjusted *p*-value (*q*-value) of ≤5%. The horizontal line on each plot represents the *q*-value cut-off. Figure S2: Cell Line-Specific Differential Gene Expression Profiles. Agglomerative hierarchically clustered heatmaps of mean-centered, TMM normalised log_2_-transformed CPM gene expression values as quantified by mRNA-Seq for Vybrant^®^ DiD−negative (DiD−) and Vybrant^®^ DiD−positive (DiD+) sub-populations within MCF-7 (A), SK-BR-3 (B), MDA-MB-468 (C) and MDA-MB-231 (D) human breast cancer cell lines. Clustering is unsupervised and based on Euclidean distances calculated from an average-linkage matrix. The top 50 most highly differentially expressed genes with a log_2_ fold-change significantly above 1.5 (up-regulated) or below −1.5 (down-regulated) at an FDR-adjusted *p*-value (*q*-value) ≤ 5% are shown. Grey and red bars represent DiD− and DiD+ grouping, respectively. Figure S3: Analysis of the Quiescent Breast Cancer Cell Transcriptome. (A) Agglomerative hierarchically clustered heatmap of MSigDB hallmark collection gene ontologies according to significance of enrichment as determined by gene set enrichment analysis. Values shown within cells are enrichment score normalised to mean enrichment of randomised equivalent samples (hypergeometric test with post hoc Benjamini-Hochberg FDR adjustment). For clarity, only statistically significant values are shown. Clustering of cell lines and gene sets is unsupervised and based on Euclidean distances calculated from an average-linkage matrix. (B) Comparison of the average clustering coefficient measured for the quiescence-associated functional association network and random networks generated from 10^4^ degree-preserving rounds of edge rewiring (two-tailed one-sample Z-test, **** = *p* ≤ 0.0001). (C) Gene set enrichment plot for inferred members of the quiescence-associated functional association network. Maximal enrichment score (ES = 0.696, dashed horizontal red line) and leading-edge genes (n = 32, dashed vertical blue line) contributing to the enrichment score are indicated. (D) Cox proportional hazards survival analysis of the quiescence-associated signature genes represented in primary breast tumours of patients in the CB482 composite data set (n = 482). Patients were stratified at all possible cut-off points (n−1) for all signature genes. For each gene, the log_2_-transformed cumulative hazard ratio for all cut-off points significantly (*p* ≤ 0.05) associated with late distant metastatic recurrence and early distant metastatic recurrence (<5 and ≥5 years after primary tumour diagnosis, respectively), normalised to total possible cut-off points, are shown. Quiescence-associated signature genes were significantly associated with late metastatic recurrence (Wilcoxon rank-sum test, **** = *p* ≤ 0.0001). Table S1, Quiescence-Associated Gene Network, Table S2: Clinical Data Set Summary Statistics.

## References

[1] Klein C.A. (2020). Cancer progression and the invisible phase of metastatic colonization. Nat. Rev. Cancer.

[2] Gomis R.R., Gawrzak S. (2017). Tumor cell dormancy. Mol. Oncol..

[3] Early Breast Cancer Trialists’ Collaborative Group (2005). Effects of chemotherapy and hormonal therapy for early breast cancer on recurrence and 15-year survival: An overview of the randomised trials. Lancet.

[4] Hess K.R., Pusztai L., Buzdar A.U., Hortobagyi G.N. (2003). Estrogen receptors and distinct patterns of breast cancer relapse. Breast Cancer Res. Treat..

[5] Goss P.E., Chambers A.F. (2010). Does tumour dormancy offer a therapeutic target?. Nat. Rev. Cancer.

[6] Nik Nabil W.N., Xi Z., Song Z., Jin L., Zhang X.D., Zhou H., De Souza P., Dong Q., Xu H. (2021). Towards a Framework for Better Understanding of Quiescent Cancer Cells. Cells.

[7] Naumov G.N., Townson J.L., MacDonald I.C., Wilson S.M., Bramwell V.H., Groom A.C., Chambers A.F. (2003). Ineffectiveness of doxorubicin treatment on solitary dormant mammary carcinoma cells or late-developing metastases. Breast Cancer Res. Treat..

[8] Quayle L.A., Ottewell P.D., Holen I. (2018). Chemotherapy resistance and stemness in mitotically quiescent human breast cancer cells identified by fluorescent dye retention. Clin. Exp. Metastasis.

[9] Giancotti F.G. (2013). Mechanisms governing metastatic dormancy and reactivation. Cell.

[10] Pan H., Gray R., Braybrooke J., Davies C., Taylor C., McGale P., Peto R., Pritchard K.I., Bergh J., Dowsett M. (2017). 20-Year Risks of Breast-Cancer Recurrence after Stopping Endocrine Therapy at 5 Years. N. Engl. J. Med..

[11] Montagner M., Sahai E. (2020). In vitro Models of Breast Cancer Metastatic Dormancy. Front. Cell Dev. Biol..

[12] Risson E., Nobre A.R., Maguer-Satta V., Aguirre-Ghiso J.A. (2020). The current paradigm and challenges ahead for the dormancy of disseminated tumor cells. Nat. Cancer.

[13] Deleyrolle L.P., Harding A., Cato K., Siebzehnrubl F.A., Rahman M., Azari H., Olson S., Gabrielli B., Osborne G., Vescovi A. (2011). Evidence for label-retaining tumour-initiating cells in human glioblastoma. Brain A J. Neurol..

[14] Dembinski J.L., Krauss S. (2009). Characterization and functional analysis of a slow cycling stem cell-like subpopulation in pancreas adenocarcinoma. Clin. Exp. Metastasis.

[15] Kusumbe A.P., Bapat S.A. (2009). Cancer stem cells and aneuploid populations within developing tumors are the major determinants of tumor dormancy. Cancer Res..

[16] Moore N., Houghton J., Lyle S. (2012). Slow-cycling therapy-resistant cancer cells. Stem Cells Dev..

[17] Roesch A., Fukunaga-Kalabis M., Schmidt E.C., Zabierowski S.E., Brafford P.A., Vultur A., Basu D., Gimotty P., Vogt T., Herlyn M. (2010). A temporarily distinct subpopulation of slow-cycling melanoma cells is required for continuous tumor growth. Cell.

[18] Wang N., Docherty F., Brown H.K., Reeves K., Fowles A., Lawson M., Ottewell P.D., Holen I., Croucher P.I., Eaton C.L. (2015). Mitotic quiescence, but not unique “stemness”, marks the phenotype of bone metastasis-initiating cells in prostate cancer. FASEB J. Off. Publ. Fed. Am. Soc. Exp. Biol..

[19] Yumoto K., Berry J.E., Taichman R.S., Shiozawa Y. (2014). A novel method for monitoring tumor proliferation in vivo using fluorescent dye DiD. Cytometry. Part A J. Int. Soc. Anal. Cytol..

[20] Andrews S. (2010). FastQC: A Quality Control Tool for High Throughput Sequence Data. http://wwwbioinformaticsbabrahamacuk/projects/fastqc/.

[21] Martin M. (2011). Cutadapt removes adapter sequences from high-throughput sequencing reads. EMBnet. J..

[22] Dobin A., Davis C.A., Schlesinger F., Drenkow J., Zaleski C., Jha S., Batut P., Chaisson M., Gingeras T.R. (2012). STAR: Ultrafast universal RNA-seq aligner. Bioinformatics.

[23] Li B., Dewey C.N. (2011). RSEM: Accurate transcript quantification from RNA-Seq data with or without a reference genome. BMC Bioinform..

[24] Lun A.T., Chen Y., Smyth G.K. (2016). It’s DE-licious: A Recipe for Differential Expression Analyses of RNA-seq Experiments Using Quasi-Likelihood Methods in edgeR. Methods Mol. Biol..

[25] Korotkevich G., Sukhov V., Budin N., Shpak B., Artyomov M.N., Sergushichev A. (2021). Fast gene set enrichment analysis. bioRxiv.

[26] Dolgalev I. (2020). msigdbr: MSigDB Gene Sets for Multiple Organisms in a Tidy Data Format. https://CRANR-projectorg/package=msigdbr.

[27] Csardi G., Nepusz T. (2006). The igraph software package for complex network research. Complex Syst..

[28] Warde-Farley D., Donaldson S.L., Comes O., Zuberi K., Badrawi R., Chao P., Franz M., Grouios C., Kazi F., Lopes C.T. (2010). The GeneMANIA prediction server: Biological network integration for gene prioritization and predicting gene function. Nucleic Acids Res..

[29] Snel B., Lehmann G., Bork P., Huynen M.A. (2000). STRING: A web-server to retrieve and display the repeatedly occurring neighbourhood of a gene. Nucleic Acids Res..

[30] Davis S., Meltzer P.S. (2007). GEOquery: A bridge between the Gene Expression Omnibus (GEO) and BioConductor. Bioinformatics.

[31] Jacobsen A., Luna A. (2019). cgdsr: R-Based API for Accessing the MSKCC Cancer Genomics. Data Server (CGDS). https://cran.r-project.org/web/packages/cgdsr/cgdsr.pdf.

[32] Leek J.T., Johnson W.E., Parker H.S., Fertig E.J., Jaffe A.E., Zhang Y., Storey J.D., Torres L.C. (2020). sva: Surrogate Variable Analysis. https://bioconductor.org/packages/release/bioc/html/sva.html.

[33] Li Q., Birkbak N.J., Gyorffy B., Szallasi Z., Eklund A.C. (2011). Jetset: Selecting the optimal microarray probe set to represent a gene. BMC Bioinform..

[34] Therneau T.M. (2021). A Package for Survival Analysis in R.

[35] Kassambara A., Kosinski M., Biecek P., Fabian S. (2020). survminer: Drawing Survival Curves Using ‘ggplot2’. https://cran.r-project.org/web/packages/survminer/survminer.pdf.

[36] Pearce D.A., Nirmal A.J., Freeman T.C., Sims A.H. (2018). Continuous Biomarker Assessment by Exhaustive Survival Analysis. bioRxiv.

[37] Eyre R., Alférez D.G., Santiago-Gómez A., Spence K., McConnell J.C., Hart C., Simões B.M., Lefley D., Tulotta C., Storer J. (2019). Microenvironmental IL1β promotes breast cancer metastatic colonisation in the bone via activation of Wnt signalling. Nat. Commun..

[38] Gao H., Chakraborty G., Lee-Lim A.P., Mo Q., Decker M., Vonica A., Shen R., Brogi E., Brivanlou A.H., Giancotti F.G. (2012). The BMP inhibitor Coco reactivates breast cancer cells at lung metastatic sites. Cell.

[39] Kim R.S., Avivar-Valderas A., Estrada Y., Bragado P., Sosa M.S., Aguirre-Ghiso J.A., Segall J.E. (2012). Dormancy signatures and metastasis in estrogen receptor positive and negative breast cancer. PLoS ONE.

[40] Park S.B., Hwang K.T., Chung C.K., Roy D., Yoo C. (2020). Causal Bayesian gene networks associated with bone, brain and lung metastasis of breast cancer. Clin. Exp. Metastasis.

[41] Sarvi S., Patel H., Li J., Dodd G.L., Creedon H., Muir M., Ward J., Dawson J.C., Lee M., Culley J. (2018). Kindlin-1 Promotes Pulmonary Breast Cancer Metastasis. Cancer Res..

[42] Kenny P.A., Lee G.Y., Myers C.A., Neve R.M., Semeiks J.R., Spellman P.T., Lorenz K., Lee E.H., Barcellos-Hoff M.H., Petersen O.W. (2007). The morphologies of breast cancer cell lines in three-dimensional assays correlate with their profiles of gene expression. Mol. Oncol..

[43] Almog N., Ma L., Raychowdhury R., Schwager C., Erber R., Short S., Hlatky L., Vajkoczy P., Huber P.E., Folkman J. (2009). Transcriptional switch of dormant tumors to fast-growing angiogenic phenotype. Cancer Res..

[44] Schmidt M., Lu Y., Parant J.M., Lozano G., Bacher G., Beckers T., Fan Z. (2001). Differential roles of p21(Waf1) and p27(Kip1) in modulating chemosensitivity and their possible application in drug discovery studies. Mol. Pharmacol..

[45] Sosa M.S., Bragado P., Aguirre-Ghiso J.A. (2014). Mechanisms of disseminated cancer cell dormancy: An awakening field. Nat. Rev. Cancer.

[46] Khalil B.D., Sanchez R., Rahman T., Rodriguez-Tirado C., Moritsch S., Martinez A.R., Miles B., Farias E., Mezei M., Cheung J.F. (2021). A specific agonist of the orphan nuclear receptor NR2F1 suppresses metastasis through the induction of cancer cell dormancy. bioRxiv.

[47] Aguirre-Ghiso J.A., Sosa M.S. (2018). Emerging Topics on Disseminated Cancer Cell Dormancy and the Paradigm of Metastasis. Annu. Rev. Cancer Biol..

[48] Ginestier C., Hur M.H., Charafe-Jauffret E., Monville F., Dutcher J., Brown M., Jacquemier J., Viens P., Kleer C.G., Liu S. (2007). ALDH1 is a marker of normal and malignant human mammary stem cells and a predictor of poor clinical outcome. Cell Stem Cell.

[49] Li W., Ma H., Zhang J., Zhu L., Wang C., Yang Y. (2017). Unraveling the roles of CD44/CD24 and ALDH1 as cancer stem cell markers in tumorigenesis and metastasis. Sci. Rep..

[50] Pece S., Tosoni D., Confalonieri S., Mazzarol G., Vecchi M., Ronzoni S., Bernard L., Viale G., Pelicci P.G., Di Fiore P.P. (2010). Biological and molecular heterogeneity of breast cancers correlates with their cancer stem cell content. Cell.

[51] Gerratana L., Fanotto V., Bonotto M., Bolzonello S., Minisini A.M., Fasola G., Puglisi F. (2015). Pattern of metastasis and outcome in patients with breast cancer. Clin. Exp. Metastasis.

[52] Aguirre-Ghiso J.A., Estrada Y., Liu D., Ossowski L. (2003). ERK(MAPK) activity as a determinant of tumor growth and dormancy; regulation by p38(SAPK). Cancer Res..

[53] Bragado P., Estrada Y., Parikh F., Krause S., Capobianco C., Farina H.G., Schewe D.M., Aguirre-Ghiso J.A. (2013). TGF-β2 dictates disseminated tumour cell fate in target organs through TGF-β-RIII and p38α/β signalling. Nat. Cell. Biol..

[54] Cackowski F.C., Eber M.R., Rhee J., Decker A.M., Yumoto K., Berry J.E., Lee E., Shiozawa Y., Jung Y., Aguirre-Ghiso J.A. (2017). Mer Tyrosine Kinase Regulates Disseminated Prostate Cancer Cellular Dormancy. J. Cell Biochem..

[55] Harper K.L., Sosa M.S., Entenberg D., Hosseini H., Cheung J.F., Nobre R., Avivar-Valderas A., Nagi C., Girnius N., Davis R.J. (2016). Mechanism of early dissemination and metastasis in Her2(+) mammary cancer. Nature.

[56] Schewe D.M., Aguirre-Ghiso J.A. (2008). ATF6alpha-Rheb-mTOR signaling promotes survival of dormant tumor cells in vivo. Proc. Natl. Acad. Sci. USA.

