# Supplementary material for: Transcriptomic Profiling Reveals Novel Candidate Genes and Signalling Programs in Breast Cancer Quiescence and Dormancy
Source: Cancers (Basel). 2021 Aug 4;13(16):3922. doi: 10.3390/cancers13163922 (PMC8392441; doi:10.3390/cancers13163922)
Supplement: Supplementary file 1 [file cancers-13-03922-s001.zip › supplementary_table_S1.pdf]

Supplementary Table S1. Quiescence-Associated Gene Network

Name	HUGO Symbol	Entrez ID	Ensembl ID	Category[a]	Sub-Network[b]	Log_2 Fold-Change[c]	DE q-value[d]	Centrality Score[e]	Metastasis Associated[f]	Quiescence or Dormancy Associated[g]
alpha-2-macroglobulin	A2M	2	ENSG00000175899	Empirical	1	1.350	0.04320	2.59	+	
ATP binding cassette subfamily C member 11	ABCC11	85320	ENSG00000121270	Empirical	17	0.932	0.00709	0.00435	+	
ATP binding cassette subfamily C member 9	ABCC9	10060	ENSG00000069431	Empirical	17	1.480	0.00687	2.02	+	
ABI family member 3 binding protein	ABI3BP	25890	ENSG00000154175	Empirical	4	1.310	0.01760	2.32	+	
actin alpha 2, smooth muscle	ACTA2	59	ENSG00000107796	Empirical	8	1.270	0.02080	0.0416	+	+
ADAM metallopeptidase domain 2	ADAM2	2515	ENSG00000104755	Empirical	13	2.440	1.54E-07	0.305	+	
ADAM metallopeptidase domain 29	ADAM29	11086	ENSG00000168594	Empirical	13	1.400	0.02000	0.00435	+	
adhesion G protein-coupled receptor F4	ADGRF4	221393	ENSG00000153294	Empirical	2	1.340	8.21E-07	0.0129		
anterior gradient 3, protein disulphide isomerase family member	AGR3	155465	ENSG00000173467	Empirical	5	1.460	1.50E-04	0.00435	+	
aldehyde dehydrogenase 1 family member A1	ALDH1A1	216	ENSG00000165092	Empirical	25	0.864	0.03250	0.00865	+	+
arachidonate 5-lipoxygenase	ALOX5	240	ENSG00000012779	Empirical	4	1.320	0.00681	1.16	+	
BPI fold containing family B member 1	BPIFB1	92747	ENSG00000125999	Empirical	28	1.950	2.75E-10	6.91E-13	+	
coiled-coil domain containing 85A	CCDC85A	114800	ENSG00000055813	Empirical	6	1.170	0.01340	0.00865	+	
C-C motif chemokine ligand 5	CCL5	6352	ENSG00000271503	Empirical	5	2.760	6.40E-07	1.73	+	+
cellular communication network factor 3	CCN3	4856	ENSG00000136999	Empirical	1	1.160	0.00313	2.37	+	
cadherin 10	CDH10	1008	ENSG00000040731	Empirical	18	1.410	0.03120	0.0171	+	
cadherin 11	CDH11	1009	ENSG00000140937	Empirical	18	0.924	0.00101	0.0171	+	+
cadherin 5	CDH5	1003	ENSG00000179776	Empirical	18	1.280	0.01090	0.0171	+	+
CEA cell adhesion molecule 5	CEACAM5	1048	ENSG00000105388	Empirical	14	1.710	3.95E-07	0.00435	+	+
complement factor B	CFB	629	ENSG00000243649	Empirical	1	0.964	0.02140	2.31	+	
chitinase 3 like 1	CHI3L1	1116	ENSG00000133048	Empirical	1	1.880	5.09E-04	0.00435	+	+
contactin 3	CNTN3	5067	ENSG00000113805	Empirical	14	1.340	0.01260	0.305		
collagen type XVII alpha 1 chain	COL17A1	1308	ENSG00000065618	Empirical	4	1.160	0.00267	2.02	+	
carboxypeptidase Z	CPZ	8532	ENSG00000109625	Empirical	2	2.020	8.76E-07	0.00435	+	
corticotropin releasing hormone receptor 1	CRHR1	1394	ENSG00000120088	Empirical	2	1.340	0.03060	0.0171	+	
cysteine rich secretory protein LCCL domain containing 1	CRISPLD1	83690	ENSG00000121005	Empirical	21	-1.090	5.04E-04	0.00435		
cystatin SN	CST1	1469	ENSG00000170373	Empirical	12	2.640	6.16E-13	0.0129	+	+
cystatin SA	CST2	1470	ENSG00000170369	Empirical	12	2.010	9.75E-06	0.00865	+	
cystatin S	CST4	1472	ENSG00000101441	Empirical	12	1.910	3.91E-10	0.0129	+	
C-X-C motif chemokine ligand 11	CXCL11	6373	ENSG00000169248	Empirical	5	1.750	0.00386	1.72	+	+
C-X-C motif chemokine ligand 14	CXCL14	9547	ENSG00000145824	Empirical	5	1.620	2.40E-04	0.00865	+	
cytochrome P450 family 1 subfamily A member 1	CYP1A1	1543	ENSG00000140465	Empirical	25	1.240	0.00207	0.00865	+	
ectodysplasin A2 receptor	EDA2R	60401	ENSG00000131080	Empirical	16	1.560	3.31E-05	0.00435	+	
ectonucleoside triphosphate diphosphohydrolase 3	ENTPD3	956	ENSG00000168032	Empirical	27	0.958	0.02600	0.00435	+	
endogenous retrovirus group W member 1, envelope	ERVW-1	30816	ENSG00000242950	Empirical	9	1.930	7.46E-05	0.00865		
fibroblast growth factor 13	FGF13	2258	ENSG00000129682	Empirical	10	1.040	0.03260	0.00435	+	
fibromodulin	FMOD	2331	ENSG00000122176	Empirical	1	1.500	0.00648	0.00435	+	
formyl peptide receptor 3	FPR3	2359	ENSG00000187474	Empirical	5	2.950	3.43E-08	2.49	+	
guanylate binding protein 4	GBP4	115361	ENSG00000162654	Empirical	3	1.320	0.00124	0.0296	+	
glucagon like peptide 2 receptor	GLP2R	9340	ENSG00000065325	Empirical	2	1.460	0.04470	0.0171		
glutamate ionotropic receptor kainate type subunit 2	GRIK2	2898	ENSG00000164418	Empirical	20	1.200	0.01340	0.0171	+	
guanylate cyclase 1 soluble subunit alpha 2	GUCY1A2	2977	ENSG00000152402	Empirical	27	-1.190	0.01650	0.00435	+	
HCK proto-oncogene, Src family tyrosine kinase	HCK	3055	ENSG00000101336	Empirical	2	1.460	0.00137	1.85	+	
major histocompatibility complex, class II, DQ beta 1	HLA-DQB1	3119	ENSG00000179344	Empirical	3	1.100	0.00707	1.86	+	
major histocompatibility complex, class II, DR beta 1	HLA-DRB1	3123	ENSG00000196126	Empirical	3	0.881	0.00857	2.22	+	
3-hydroxy-3-methylglutaryl-CoA synthase 2	HMGCS2	3158	ENSG00000134240	Empirical	23	1.370	8.05E-04	0.00435	+	
interferon induced protein with tetratricopeptide repeats 2	IFIT2	3433	ENSG00000119922	Empirical	3	1.210	0.00379	2.02	+	
insulin like growth factor binding protein 5	IGFBP5	3488	ENSG00000115461	Empirical	1	1.120	0.00392	2.8	+	
janus kinase and microtubule interacting protein 2	JAKMIP2	9832	ENSG00000176049	Empirical	15	2.080	2.38E-05	0.00865		
potassium inwardly rectifying channel subfamily J member 8	KCNJ8	3764	ENSG00000121361	Empirical	17	1.540	0.00100	2.32		
kallikrein related peptidase 11	KLK11	11012	ENSG00000167757	Empirical	1	2.530	2.23E-06	1.28	+	
kallikrein related peptidase 8	KLK8	11202	ENSG00000129455	Empirical	1	1.340	7.97E-05	1.28	+	
keratin 16	KRT16	3868	ENSG00000186832	Empirical	6	1.180	2.38E-05	0.957	+	
laminin subunit gamma 2	LAMC2	3918	ENSG00000058085	Empirical	4	1.030	0.00379	3.09	+	+
leucine rich repeat neuronal 1	LRRN1	57633	ENSG00000175928	Empirical	14	1.250	0.00583	0.00435	+	
maltase-glucoamylase	MGAM	8972	ENSG00000257335	Empirical	1	1.390	0.04280	2.04	+	
mucin 5AC, oligomeric mucus/gel-forming	MUC5AC	4586	ENSG00000215182	Empirical	1	1.420	3.80E-04	2.02	+	+
mucin like 1	MUCL1	118430	ENSG00000172551	Empirical	1	1.370	0.00101	0.00435	+	
neutrophil cytosolic factor 2	NCF2	4688	ENSG00000116701	Empirical	3	1.500	1.34E-06	0.00435	+	
2'-5'-oligoadenylate synthetase 2	OAS2	4939	ENSG00000111335	Empirical	3	0.968	8.05E-04	1.57	+	
2'-5'-oligoadenylate synthetase like	OASL	8638	ENSG00000135114	Empirical	3	1.150	0.02300	2.82		
peptidase inhibitor 16	PI16	221476	ENSG00000164530	Empirical	21	2.230	2.65E-07	0.00435	+	
plasminogen activator, tissue type	PLAT	5327	ENSG00000104368	Empirical	1	1.050	0.03120	2.39	+	
phospholipase C like 1 (inactive)	PLCL1	5334	ENSG00000115896	Empirical	11	1.280	0.00774	0.0129	+	
protein phosphatase 1 regulatory inhibitor subunit 1A	PPP1R1A	5502	ENSG00000135447	Empirical	19	1.510	0.00508	0.305	+	
serine protease 2	PRSS2	5645	ENSG00000275896	Empirical	1	1.540	2.00E-04	1.79	+	
prostaglandin F receptor	PTGFR	5737	ENSG00000122420	Empirical	5	1.230	0.00476	2.32	+	
prostaglandin-endoperoxide synthase 2	PTGS2	5743	ENSG00000073756	Empirical	4	1.430	0.02220	2.23	+	
protein tyrosine phosphatase receptor type H	PTPRH	5794	ENSG00000080031	Empirical	4	1.070	0.00107	2.49	+	
protein tyrosine phosphatase receptor type N2	PTPRN2	5799	ENSG00000155093	Empirical	4	1.880	2.86E-05	2.61	+	
radical S-adenosyl methionine domain containing 2	RSAD2	91543	ENSG00000134321	Empirical	3	1.770	8.21E-07	0.0213	+	
somatomedin B and thrombospondin type 1 domain containing	SBSPON	157869	ENSG00000164764	Empirical	1	1.570	0.01280	0.0129		
secretoglobin family 1D member 2	SCGB1D2	10647	ENSG00000124935	Empirical	22	1.710	0.00642	0.00865	+	+
secretoglobin family 2A member 2	SCGB2A2	4250	ENSG00000110484	Empirical	22	2.140	3.74E-06	0.00865	+	+
sodium voltage-gated channel alpha subunit 3	SCN3A	6328	ENSG00000153253	Empirical	10	1.380	0.04320	0.703	+	
signal peptide, CUB domain and EGF like domain containing 2	SCUBE2	57758	ENSG00000175356	Empirical	7	0.893	0.03800	2.24	+	
semaphorin 5B	SEMA5B	54437	ENSG00000082684	Empirical	1	2.460	7.93E-07	0.0129	+	
serpin family A member 1	SERPINA1	5265	ENSG00000197249	Empirical	1	1.200	7.93E-06	2.93	+	
serpin family A member 3	SERPINA3	12	ENSG00000196136	Empirical	1	1.260	5.13E-04	0.00865	+	
serpin family E member 1	SERPINE1	5054	ENSG00000106366	Empirical	1	1.420	0.01340	2.93	+	
sonic hedgehog signaling molecule	SHH	6469	ENSG00000164690	Empirical	2	0.885	0.01030	2.84	+	+
sialic acid binding Ig like lectin 6	SIGLEC6	946	ENSG00000105492	Empirical	3	0.883	0.02220	0.00865		
solute carrier family 14 member 1 (Kidd blood group)	SLC14A1	6563	ENSG00000141469	Empirical	30	1.120	0.03150	1.85E-14	+	
solute carrier family 1 member 1	SLC1A1	6505	ENSG00000106688	Empirical	9	0.916	0.00836	0.605	+	
solute carrier family 34 member 2	SLC34A2	10568	ENSG00000157765	Empirical	26	1.140	0.00224	0.00435	+	
superoxide dismutase 3	SOD3	6649	ENSG00000109610	Empirical	7	0.988	1.02E-04	2.32	+	+
small proline rich protein 3	SPRR3	6707	ENSG00000163209	Empirical	24	2.320	8.05E-04	0.00435	+	
synaptotagmin 10	SYT10	341359	ENSG00000110975	Empirical	11	1.110	0.01230	0.0129		
synaptotagmin 5	SYT5	6861	ENSG00000129990	Empirical	11	1.380	0.00147	0.0171		
trefoil factor 3	TFF3	7033	ENSG00000160180	Empirical	2	0.981	1.43E-04	1.84	+	+
TNF receptor superfamily member 10c	TNFRSF10C	8794	ENSG00000173535	Empirical	16	1.140	0.01340	2.02	+	
tenascin XB	TNXB	7148	ENSG00000168477	Empirical	4	1.040	0.00163	2.02	+	
tyrosinase related protein 1	TYRP1	7306	ENSG00000107165	Empirical	31	1.390	0.04930	3.09E-14	+	
uroplakin 1A	UPK1A	11045	ENSG00000105668	Empirical	4	1.510	8.03E-04	2.02	+	
uroplakin 3A	UPK3A	7380	ENSG00000100373	Empirical	29	1.680	2.90E-04	0	+	
von Willebrand factor A domain containing 5A	VWA5A	4013	ENSG00000110002	Empirical	1	1.410	0.00153	0.00435		
zinc finger DHHC-type palmitoyltransferase 22	ZDHHC22	283576	ENSG00000177108	Empirical	2	1.170	0.00709	0.00435		
ADP ribosylation factor like GTPase 6 interacting protein 5	ARL6IP5	10550	ENSG00000144746	Inferred	9	0.063	1.00000	0.00435	+	
aryl hydrocarbon receptor nuclear translocator like 2	ARNTL2	56938	ENSG00000029153	Inferred	1	0.057	1.00000	0.0171	+	
complement C3	C3	718	ENSG00000125730	Inferred	1	0.037	1.00000	3	+	+
Cbl proto-oncogene	CBL	867	ENSG00000110395	Inferred	2	-0.002	1.00000	2.52	+	
coiled-coil domain containing 85B	CCDC85B	11007	ENSG00000175602	Inferred	6	-0.084	1.00000	0.956	+	
coiled-coil domain containing 85C	CCDC85C	317762	ENSG00000205476	Inferred	6	-0.076	1.00000	0.00865	+	
C-C motif chemokine ligand 20	CCL20	6364	ENSG00000115009	Inferred	5	0.674	1.00000	0.0255	+	+
copper chaperone for superoxide dismutase	CCS	9973	ENSG00000173992	Inferred	7	0.050	1.00000	0.0129	+	
CD151 molecule (Raph blood group)	CD151	977	ENSG00000177697	Inferred	4	0.101	1.00000	2.32	+	
CD74 molecule	CD74	972	ENSG00000019582	Inferred	3	0.654	1.00000	0.0495	+	+
cyclin dependent kinase inhibitor 1A	CDKN1A	1026	ENSG00000124762	Inferred	2	0.648	1.00000	0.0296	+	+
complement factor D	CFD	1675	ENSG00000197766	Inferred	1	0.075	1.00000	2.57	+	
complement factor properdin	CFP	5199	ENSG00000126759	Inferred	1	0.517	1.00000	2.37	+	+
calmegin	CLGN	1047	ENSG00000153132	Inferred	13	0.480	1.00000	0.00435	+	
clock circadian regulator	CLOCK	9575	ENSG00000134852	Inferred	1	-0.033	1.00000	0.0171	+	+
CRK like proto-oncogene, adaptor protein	CRKL	1399	ENSG00000099942	Inferred	2	-0.035	1.00000	0.0377	+	
derlin 2	DERL2	51009	ENSG00000072849	Inferred	2	-0.108	1.00000	2.82		
epidermal growth factor receptor	EGFR	1956	ENSG00000146648	Inferred	2	0.291	1.00000	3.3	+	+
fibulin 5	FBLN5	10516	ENSG00000140092	Inferred	7	0.373	1.00000	2.6	+	
glutamate ionotropic receptor kainate type subunit 4	GRIK4	2900	ENSG00000149403	Inferred	20	0.028	1.00000	0.0129		
glutamate ionotropic receptor kainate type subunit 5	GRIK5	2901	ENSG00000105737	Inferred	20	0.037	1.00000	0.0129		
hedgehog interacting protein	HHIP	64399	ENSG00000164161	Inferred	2	0.402	1.00000	0.00866	+	
major histocompatibility complex, class II, DM alpha	HLA-DMA	3108	ENSG00000204257	Inferred	3	0.299	1.00000	0.0456	+	+

Name	HUGO Symbol	Entrez ID	Ensembl ID	Category[a]	Sub-Network[b]	Log_2 Fold-Change[c]	DE q-value[d]	Centrality Score[e]	Metastasis Associated[f]	Quiescence or Dormancy Associated[g]
major histocompatibility complex, class II, DQ alpha 1	HLA-DQA1	3117	ENSG00000196735	Inferred	3	0.169	1.00000	1.53	+	
major histocompatibility complex, class II, DR alpha	HLA-DRA	3122	ENSG00000204287	Inferred	3	0.820	0.35900	1.98	+	
major histocompatibility complex, class II, DR beta 5	HLA-DRB5	3127	ENSG00000198502	Inferred	3	0.777	0.47200	1.61	+	
3-hydroxy-3-methylglutaryl-CoA synthase 1	HMGCS1	3157	ENSG00000112972	Inferred	23	-0.062	1.00000	0.00435	+	
interferon induced protein with tetratricopeptide repeats 1	IFIT1	3434	ENSG00000185745	Inferred	3	0.699	1.00000	2.05	+	
interferon induced protein with tetratricopeptide repeats 3	IFIT3	3437	ENSG00000119917	Inferred	3	0.628	1.00000	0.0336	+	
insulin like growth factor 2	IGF2	3481	ENSG00000167244	Inferred	1	0.904	0.68100	3.07	+	+
insulin like growth factor binding protein acid labile subunit	IGFALS	3483	ENSG00000099769	Inferred	1	0.475	1.00000	2.02		
involucrin	IVL	3713	ENSG00000163207	Inferred	24	0.516	1.00000	0.00435	+	
janus kinase and microtubule interacting protein 1	JAKMIP1	152789	ENSG00000152969	Inferred	15	0.313	1.00000	0.00865		
Janus kinase and microtubule interacting protein 3	JAKMIP3	282973	ENSG00000188385	Inferred	15	-0.104	1.00000	0.00865		
keratin 10	KRT10	3858	ENSG00000186395	Inferred	6	-0.051	1.00000	0.0171	+	
keratin 5	KRT5	3852	ENSG00000186081	Inferred	6	0.372	1.00000	0.0171	+	+
ladinin 1	LAD1	3898	ENSG00000159166	Inferred	4	-0.084	1.00000	0.00435	+	
laminin subunit beta 3	LAMB3	3914	ENSG00000196878	Inferred	4	0.640	1.00000	0.0213	+	
myosin heavy chain 11	MYH11	4629	ENSG00000133392	Inferred	8	0.459	1.00000	0.0416	+	
2'-5'-oligoadenylate synthetase 1	OAS1	4938	ENSG00000089127	Inferred	3	0.901	0.06540	0.0129	+	
2'-5'-oligoadenylate synthetase 3	OAS3	4940	ENSG00000111331	Inferred	3	0.193	1.00000	0.0129		
phosphatidylinositol-4,5-bisphosphate 3-kinase catalytic subunit beta	PIK3CB	5291	ENSG00000051382	Inferred	2	-0.057	1.00000	2.49	+	
plasminogen activator, urokinase	PLAU	5328	ENSG00000122861	Inferred	1	0.604	1.00000	2.55	+	+
phospholipase C gamma 1	PLCG1	5335	ENSG00000124181	Inferred	3	-0.057	1.00000	2.8	+	
plectin	PLEC	5339	ENSG00000178209	Inferred	4	0.107	1.00000	2.77	+	
protein phosphatase 1 regulatory subunit 15A	PPP1R15A	23645	ENSG00000087074	Inferred	19	0.221	1.00000	0.00435	+	
protein phosphatase 1 regulatory inhibitor subunit 1C	PPP1R1C	151242	ENSG00000150722	Inferred	19	0.830	1.00000	0.00435	+	
patched 1	PTCH1	5727	ENSG00000185920	Inferred	2	0.185	1.00000	2.49	+	
patched 2	PTCH2	8643	ENSG00000117425	Inferred	2	0.057	1.00000	0.0129	+	
prostaglandin E synthase 2	PTGES2	80142	ENSG00000148334	Inferred	5	-0.155	1.00000	2.02		
ribosomal protein S27a	RPS27A	6233	ENSG00000143947	Inferred	2	0.028	1.00000	2.72	+	
sodium voltage-gated channel alpha subunit 2	SCN2A	6326	ENSG00000136531	Inferred	10	0.486	1.00000	0.0171	+	
sodium voltage-gated channel alpha subunit 8	SCN8A	6334	ENSG00000196876	Inferred	10	0.840	1.00000	0.604	+	
sodium voltage-gated channel alpha subunit 9	SCN9A	6335	ENSG00000169432	Inferred	10	0.592	1.00000	0.0171	+	
signal peptide, CUB domain and EGF like domain containing 1	SCUBE1	80274	ENSG00000159307	Inferred	7	-0.190	1.00000	0.0129	+	
solute carrier family 1 member 4	SLC1A4	6509	ENSG00000115902	Inferred	9	-0.059	1.00000	0.308		
solute carrier family 1 member 5	SLC1A5	6510	ENSG00000105281	Inferred	9	-0.072	1.00000	0.308	+	+
solute carrier family 34 member 3	SLC34A3	142680	ENSG00000198569	Inferred	26	0.621	1.00000	0.00435		
smoothened, frizzled class receptor	SMO	6608	ENSG00000128602	Inferred	2	0.417	1.00000	2.61	+	+
superoxide dismutase 1	SOD1	6647	ENSG00000142168	Inferred	7	-0.081	1.00000	0.0129	+	
SOS Ras/Rac guanine nucleotide exchange factor 1	SOS1	6654	ENSG00000115904	Inferred	2	0.073	1.00000	2.53	+	
spectrin beta, non-erythrocytic 4	SPTBN4	57731	ENSG00000160460	Inferred	4	0.339	1.00000	2.7		
synaptotagmin 3	SYT3	84258	ENSG00000213023	Inferred	11	0.137	1.00000	0.0171		
trichoplein keratin filament binding	TCHP	84260	ENSG00000139437	Inferred	6	-0.149	1.00000	1	+	
TNF superfamily member 10	TNFSF10	8743	ENSG00000121858	Inferred	16	0.469	1.00000	2.32	+	+
tropomyosin 1	TPM1	7168	ENSG00000140416	Inferred	8	0.043	1.00000	0.0416	+	
tropomyosin 2	TPM2	7169	ENSG00000198467	Inferred	8	0.047	1.00000	0.0416	+	
tropomyosin 3	TPM3	7170	ENSG00000143549	Inferred	8	-0.146	1.00000	0.0416	+	
tropomyosin 4	TPM4	7171	ENSG00000167460	Inferred	8	-0.027	1.00000	0.0416	+	
ubiquitin A-52 residue ribosomal protein fusion product 1	UBA52	7311	ENSG00000221983	Inferred	2	-0.007	1.00000	2.6	+	
ubiquitin B	UBB	7314	ENSG00000170315	Inferred	2	-0.039	1.00000	2.21	+	
ubiquitin C	UBC	7316	ENSG00000150991	Inferred	2	0.147	1.00000	2.21	+	
uroplakin 2	UPK2	7379	ENSG00000110375	Inferred	4	0.696	1.00000	0.00435	+	
vascular endothelial growth factor B	VEGFB	7423	ENSG00000173511	Inferred	4	0.118	1.00000	0.00435	+	
vitronectin	VTN	7448	ENSG00000109072	Inferred	1	0.367	1.00000	0.0129	+	+

[a] Gene (node) categorisation according to whether it was used as an input/query gene (empirical) or added by algorithmic inference (inferred) during network construction

[b] Sub-network community membership identified by community detection based on edge betweenness

[c] Log_2-transformed fold change in mean normalised gene transcript counts as determined by RNA-Seq (proliferating Vybrant® DiD- versus quiescent Vybrant® DiD+ sub-populations)

[d] False discovery rate adjusted p-value of differential expression according to comparison of mean normalised gene transcript counts

[e] Centrality score calculated as the log_{10}-transformed weighted sum of total degree, betweenness, eigenvalue, and closeness centrality metrics combined by multidimensional scaling

[f] Association with metastasis according to automated mining of PubMed® using the search "gene[Title/Abstract] AND metasta*[Title/Abstract]"

[g] Association with dormancy or quiescence in cancer according to automated mining of PubMed® combining the results of searches "gene[Title/Abstract] AND quiescen*[Title/Abstract] AND cancer[Title/Abstract]" or "gene[Title/Abstract] AND dorman*[Title/Abstract] AND cancer[Title/Abstract]"
