# Supplementary material for: Transcriptomic Profiling Reveals Novel Candidate Genes and Signalling Programs in Breast Cancer Quiescence and Dormancy
Source: Cancers (Basel). 2021 Aug 4;13(16):3922. doi: 10.3390/cancers13163922 (PMC8392441; doi:10.3390/cancers13163922)
Supplement: Supplementary file 1 [file cancers-13-03922-s001.zip › supplementary_table_S2.pdf]

Supplementary Table S2. Clinical Data Set Summary Statistics

Study Accession or ID	GSE2034	GSE2603	GSE12276	CB572	GSE1456	GSE6532	GSE7390	CB482
Number of Patients	286	82	204	572	159	125	198	482
ER[a] Negative Patients	77	36	73	186	40	34	64	138
ER Positive Patients	209	46	116	371	99	85	134	318
ER Negative Proportion (% of All Patients)	26.9	43.9	35.8	32.5	25.2	27.2	32.3	28.6
ER Positive Proportion (% of All Patients)	73.1	56.1	56.9	64.9	62.3	68	67.7	66
Patients with Distant Metastasis	107	27	185	319	40	28	62	130
Patients with Early Distant Metastasis[b]	93	22	172	287	34	21	36	91
Patients Late Distant Metastasis[c]	14	5	13	32	6	7	26	39
Distant Metastasis (% of All Patients)	37.4	32.9	90.7	55.8	25.2	22.4	31.3	27
Early Distant Metastasis (% of All Patients)	32.5	26.8	84.3	50.2	21.4	16.8	18.2	18.9
Late Distant Metastasis (% of All Patients)	4.9	6.1	6.37	5.59	3.77	5.6	13.1	8.09
Early Distant Metastasis (% Patients with Distant Metastasis)	86.9	81.5	93	90	85	75	58.1	70
Late Distant Metastasis (% Patients with Distant Metastasis)	13.1	18.5	7.03	10	15	25	41.9	30

[a] ER = Oestrogen Receptor
[b] Early distant metastasis defined as any distant recurrence event occurring <5 years from the start of follow-up time records for the study
[c] Late distant metastasis defined as any distant recurrence event occurring ≥5 years from the start of follow-up time records for the study
