# Supplementary figures and images for: Transcriptomic Profiling Reveals Novel Candidate Genes and Signalling Programs in Breast Cancer Quiescence and Dormancy
Source: Cancers (Basel). 2021 Aug 4;13(16):3922. doi: 10.3390/cancers13163922 (PMC8392441; doi:10.3390/cancers13163922)

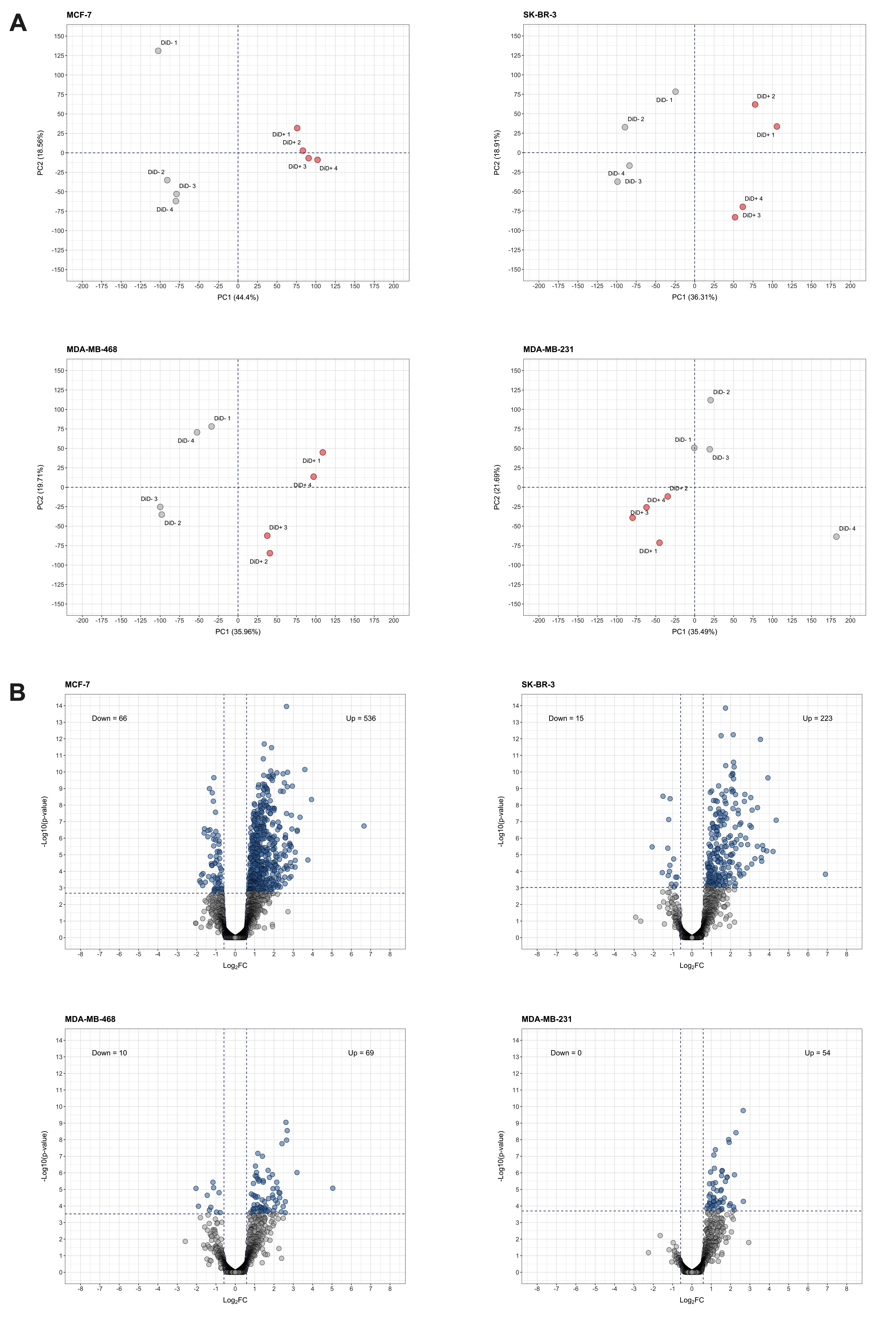

Supplement: Supplementary file 1 [file cancers-13-03922-s001.zip › supplementary_figure_S1.jpg]

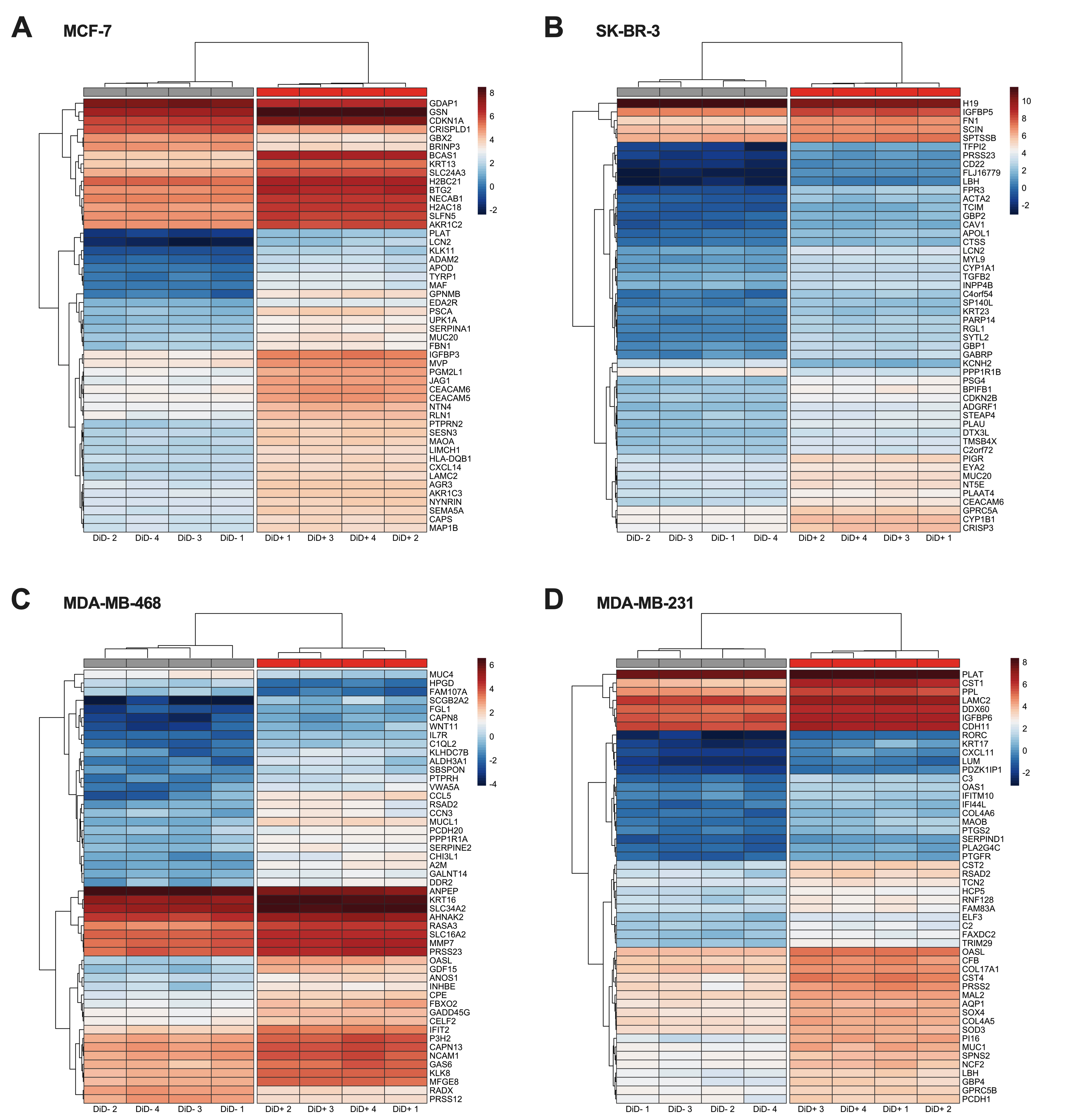

Supplement: Supplementary file 1 [file cancers-13-03922-s001.zip › supplementary_figure_S2.jpg]

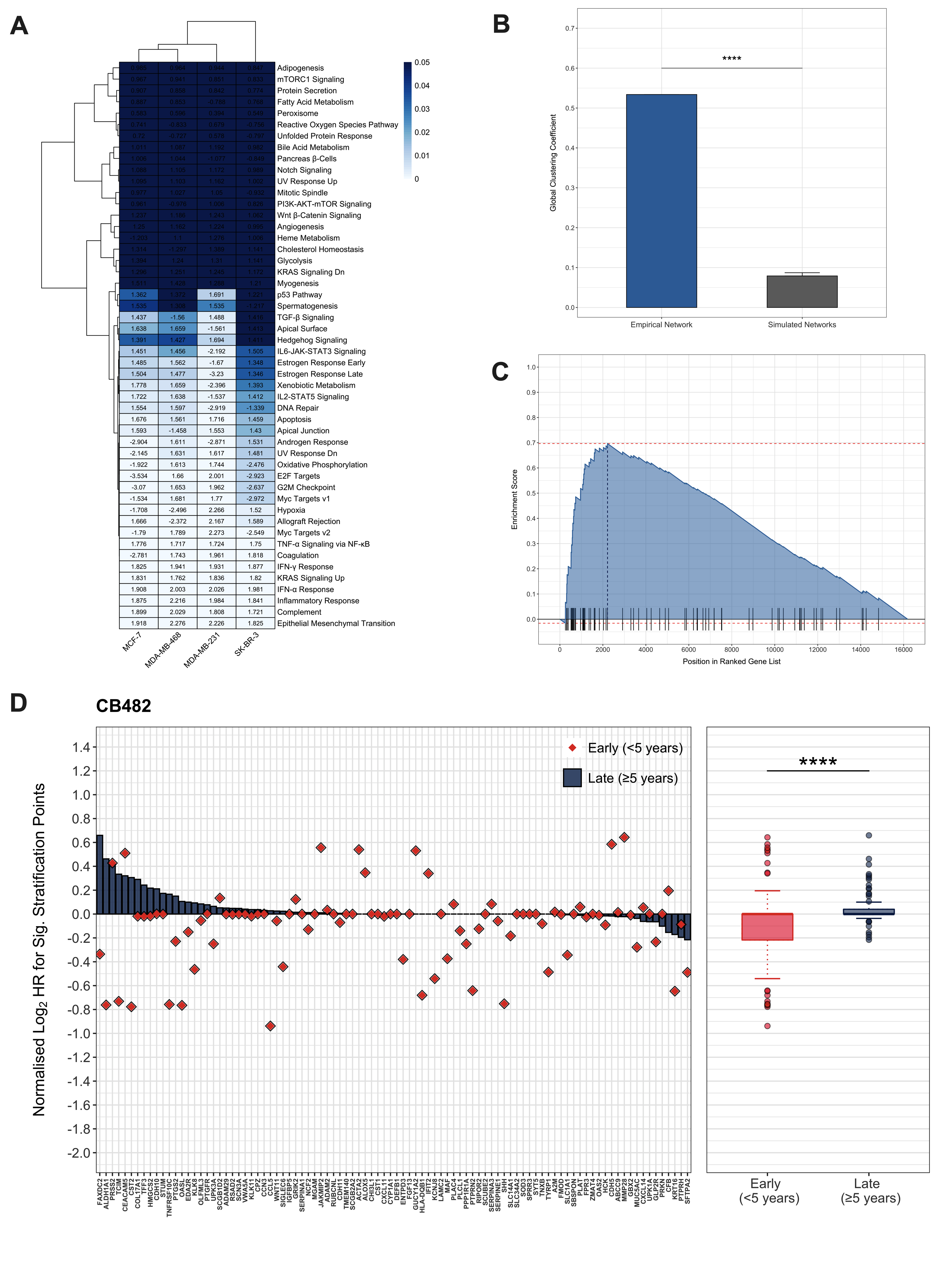

Supplement: Supplementary file 1 [file cancers-13-03922-s001.zip › supplementary_figure_S3.jpg]
